# Disrupted Intrinsic Connectivity among Default, Dorsal Attention, and Frontoparietal Control Networks in Individuals with Chronic Traumatic Brain Injury*

**DOI:** 10.1017/S1355617715001393

**Published:** 2016-02

**Authors:** Kihwan Han, Sandra B. Chapman, Daniel C. Krawczyk

**Affiliations:** 1 Center for BrainHealth^®^, School of Behavioral and Brain Sciences, The University of Texas at Dallas, Dallas, Texas; 2 Department of Psychiatry, The University of Texas Southwestern Medical Center, Dallas, Texas

**Keywords:** TBI, Functional connectivity, fMRI, Default network, Attention network, Fronto-parietal control network, Graph theory, Goal-directed cognition

## Abstract

**Objectives:** Individuals with chronic traumatic brain injury (TBI) often show detrimental deficits in higher order cognitive functions requiring coordination of multiple brain networks. Although assessing TBI-related deficits in higher order cognition in the context of network dysfunction is promising, few studies have systematically investigated altered interactions among multiple networks in chronic TBI. **Method:** We characterized disrupted resting-state functional connectivity of the default mode network (DMN), dorsal attention network (DAN), and frontoparietal control network (FPCN) whose interactions are required for internally and externally focused goal-directed cognition in chronic TBI. Specifically, we compared the network interactions of 40 chronic TBI individuals (8 years post-injury on average) with those of 17 healthy individuals matched for gender, age, and years of education. **Results:** The network-based statistic (NBS) on DMN-DAN-FPCN connectivity of these groups revealed statistically significant (*p*_NBS_<.05; |*Z|*>2.58) reductions in within-DMN, within-FPCN, DMN-DAN, and DMN-FPCN connectivity of the TBI group over healthy controls. Importantly, such disruptions occurred prominently in between-network connectivity. Subsequent analyses further exhibited the disrupted connectivity patterns of the chronic TBI group occurring preferentially in long-range and inter-hemispheric connectivity of DMN-DAN-FPCN. Most importantly, graph-theoretic analysis demonstrated relative reductions in global, local and cost efficiency (*p*<.05) as a consequence of the network disruption patterns in the TBI group. **Conclusion:** Our findings suggest that assessing multiple networks-of-interest simultaneously will allow us to better understand deficits in goal-directed cognition and other higher order cognitive phenomena in chronic TBI. Future research will be needed to better understand the behavioral consequences related to these network disruptions. (*JINS*, 2016, *22*, 263–279)

## Introduction

A traumatic brain injury (TBI) is induced by external force, leading to abnormalities in brain structure and function. Individuals with chronic TBI (6-months or more post-injury) often have difficulties in cognitive functions such as attention, memory and executive functions, which are critical to daily life tasks. One of the most relevant mechanisms of such deficits following TBI is diffuse axonal injury (DAI; Smith, Meaney, & Shull, 2003). Given the prevalence of DAI following TBI, network analyses in advanced magnetic resonance imaging (MRI) are providing new insights into the mechanisms of disruptions to the brain and associated impairments following TBI (see Sharp, Scott, & Leech 2014, for review). Several resting-state functional MRI (fMRI) studies have identified disruptions in a variety of networks following TBI (Bonnelle et al., 2011, 2012; Ham et al., 2014; Han et al., 2014; Marquez de la Plata et al., 2011; Mayer, Mannell, Ling, Gasparovic, & Yeo, 2011; Nakamura, Hillary, & Biswal, 2009; Pandit et al., 2013; Sharp et al., 2011; Slobounov et al., 2011; Sours et al., 2013, 2015; Tang et al., 2011).

Although the previous resting-state fMRI (rsfMRI) studies in TBI have provided valuable information for a better understanding of TBI, most of these previous studies have focused on investigating connectivity alterations within a single-network or patterns of connectivity for a single-brain-region with rest of the brain. For example, TBI-related disruptions of functional connectivity within the default mode network (DMN), which is “de-activated” when subjects engage in tasks (Raichle et al., 2001), have been observed in a prior study that had applied independent component analysis (ICA) (Sharp et al., 2011). Using a seed-based approach, Mayer et al. (2011) identified that TBIs can disrupt the connectivity of seed regions from the DMN and task-related network (Fox et al., 2005), respectively, with rest of the brain.

Ham et al. (2014) also revealed that impaired self-awareness in TBI is associated with reduced connectivity within the fronto-parietal control network (FPCN), which is activated during tasks that engage executive functions (Vincent, Kahn, Snyder, Raichle, & Buckner, 2008). However, probing only within-network connectivity or the connectivity of a single region with rest of the brain may not be sufficient to fully understand the neural mechanisms by which TBI disrupts brain networks leading to functional impairments, since DAI may affect multiple regions encompassing multiple networks (Han et al., 2014). These disruptions may lead to deficits of higher order cognitive functions due to reduced interactivity among multiple networks.

To more comprehensively identify TBI-related disruptions, several studies recently investigated resting-state functional connectivity over multiple networks. For example, Bonnelle et al. (2012) demonstrated that damaged white matter tracts connecting within the salience network (SN; Seeley et al., 2007) predict DMN dysfunction. Graph theoretic analyses (see Rubinov & Sporns, 2010, for review) also revealed patterns of network disruptions in TBI (Caeyenberghs et al., 2012; Caeyenberghs, Leemans, Leunissen, Michiels, & Swinnen, 2013; Han et al., 2014; Hillary et al., 2014; Nakamura et al., 2009; Pandit et al., 2013). For example, disruptions in “small-worldness”—the level of clustering relative to path length—of individuals with TBI have been reported in whole-brain functional networks (Nakamura et al., 2009). Module-based graph theoretic analyses have also been used to demonstrate prominent reductions in between-module connectivity in concussive blast-related TBI at the sub-acute stage, within 6-months post-injury (Han et al., 2014).

In this study, we assessed chronic TBI individuals’ intrinsic functional connectivity among multiple networks associated with goal-directed cognition (or goal-directed behavior). Goal-directed cognition is key in everyday life as it represents an ability to coordinate thoughts and actions to achieve goals while adjusting these goals in the context of changing task demands. Individuals with chronic TBI often exhibit difficulties in goal-directed cognition (Levine et al., 1998; Mateer, Sohlberg, & Crinean, 1987; Robertson, Manly, Andrade, Baddeley, & Yiend, 1997; Whyte et al., 1996). Impaired goal-directed cognition is associated with lowered work status (Crepeau & Scherzer, 1993), as such deficits lead to disorganization in daily life tasks such as cooking and navigating the layout of floor-plans (Levine et al., 2000).

An fMRI activation study in healthy individuals (Spreng, Stevens, Chamberlain, Gilmore, & Schacter, 2010) revealed that goal-directed cognition induces brain activations in the DMN (Raichle et al., 2001), dorsal attention network (DAN; Corbetta & Shulman, 2002), and FPCN (Vincent et al., 2008) during an autobiographical task and a visuospatial planning task [the Tower of London task (Shallice, 1982)]. Specifically, DMN regions were activated during the autobiographical task representing internally focused goal-directed cognition, whereas DAN regions were activated during the visuospatial planning task representing externally focused goal-directed cognition. Importantly, regions in the FPCN were activated during both tasks, indicating that the FPCN engages in both internally and externally focused goal-directed cognition. A follow-up study (Spreng, Sepulcre, Turner, Stevens, & Schacter, 2013) further identified that the FPCN mediates a dynamic balance between the DMN and DAN. Based on the frequent deficits in goal-directed cognition in chronic TBI and the previous literature in goal-directed cognition in healthy individuals, we hypothesized that individuals with chronic TBI would show disrupted interactions among DMN-DAN-FPCN. We predicted that an assessment of multiple networks-of-interest would provide more comprehensive insights on the brain mechanisms of the goal management deficits present in chronic TBI compared to a single network approach.

## Methods

### Participants

The data included in this analysis are part of a larger study (Krawczyk et al., 2013). We analyzed 57 individuals, comprising of 40 chronic TBI individuals with upper moderate disability to lower good recovery (age 20–45 years; >6 months post-injury; 6–7 on the Extended Glasgow Outcome Scale (GOS-E; Wilson, Pettigrew, & Teasdale, 1998) and 17 healthy controls (age 19–43 years), who completed MRI scans and whose MRI scans passed the Quality Assurance (QA) procedures described below. We recruited these participants from the Dallas area and screened via phone interview before inclusion. Not all TBI participants had an available, recorded Glasgow Coma Scale (GCS; Teasdale & Jennett, 1974) score for information on acute-injury severity. Thus, we *estimated* initial injury severity from the Ohio State University (OSU) TBI screening form (Corrigan & Bogner, 2007).

The primary causes of injury were blast, blunt force trauma, fall, athletic impacts, vehicle accidents, or combination of these events (Table 1). No TBI participants met clinical diagnostic criteria for neurological or psychiatric comorbidities. We also confirmed that all participants lacked visible focal lesions, contusions, mass shifting, or extreme cortical thinning on structural MRI scans. This step ruled out potential effects of macroscopic structural injuries on fMRI preprocessing steps including registration and subsequent functional connectivity analyses. All participants provided written informed consent, and this study was conducted in compliance with the declaration of Helsinki. This study was approved by the Institutional Review Boards of The University of Texas at Dallas and University of Texas Southwestern Medical Center.

**Table 1:** Demographics

Demographics	TBI (*N*=40)	Control (*N*=17)	*p*-Values
Age (years)^a^	31.7±6.6	27.6±8.5	0.06
Education (years)^a^	15.6±1.9	14.6±2.1	0.10
Gender (males, females)	29, 11	11, 6	0.55
Civilians, veterans	22, 18	N/A	N/A
Post-injury time (years)^a^	7.7±6.5	N/A	N/A
Estimated injury severity (mild, moderate, severe)^b^	31, 4, 5	N/A	N/A
Primary cause of injury (blast, blunt force trauma, fall, athletic impacts, vehicle accidents, combined)^b^	7, 4, 7, 8, 9, 5	N/A	N/A
BDI-II^a^	15.7±9.6	3.6±4.6	<10^-7^
PCL-S^a^	43.8±17.3	N/A^c^	N/A
Brain volume (10^6^ mm^3^)^a^	1.20±0.10	1.25±0.14	0.34^d^
Normalized brain volume (10^6^ mm^3^)^a^ ^,^ ^e^	1.61±0.07	1.65±0.08	0.20^d^
Motion-censored volumes (%)^a^	16.3±12.2	12.7±13.4	0.15
Average FD after motion censoring and trimming (mm)^a^	0.16±0.04	0.15±0.04	0.54

*Note*: FD, Framewise Displacement; BDI-II, Beck Depression Inventory-II; PCL-S, Posttraumatic Stress Disorder Check List Stressor-Specific.

a Mean and standard deviation values were reported.

b Based on the Ohio State University TBI screening form (Corrigan & Bogner, 2007).

c PCL-S scores for the healthy controls were not available since the healthy individuals, by definition, did not have a traumatic event that they are experiencing for the assessment of PCL-S.

d *p*-Values were obtained with an age covariate.

e Normalized for head size.

### Psychiatric Symptoms Assessments

We assessed symptom severity of depression and post-traumatic stress disorder (PTSD) for the participants, using the Beck Depression Inventory-II (BDI-II; Beck, Steer, & Brown, 1996) and PTSD Check List Stressor-specific (PCL-S; Weathers, Litz, Herman, Huska, & Keane, 1993). Note that we did not acquire PCL-S scores from the healthy control participants, as PCL-S requires reporting on a specific traumatic event that participant experiences. The healthy individuals, by definition, did not experience traumatic events to be reported on for the PCL-S assessment.

### Neuropsychological Assessments

We administered neuropsychological tests at the time of MRI scanning to characterize participants’ cognition in a variety of domains. These tests included similarities, matrix reasoning, and full scale intelligent quotient-2 (FSIQ-2) from the Wechsler Abbreviated Scale of Intelligence (WASI) for estimated current IQ (Wechsler, 1999); FSIQ from the Wechsler Test of Adult Reading (WTAR) for estimated premorbid IQ (Wechsler, 2001); digit span forward and backward from the Wechsler Adult Intelligence Scale-Third Edition (WAIS-III) for working memory (Wechsler, 1997); color-word, verbal fluency, card sorting, trail making from the Delis-Kaplan Executive Function System (D-KEFS) for measuring inhibitory control, switching, verbal fluency, processing speed and problem solving (Delis, Kaplan, & Kramer, 2001); immediate recall and delayed recall from the Wechsler Memory Scale-Fourth Edition (WMS-IV) for memory and recall (Wechsler, 2008); verbal problem solving assessment (S.B. Chapman, unpublished data); and visual selective learning task adapted from Hanten et al. (Hanten et al., 2004). We also assessed the satisfaction with life scale (Diener, Emmons, Larsen, & Griffin, 1985) to measure global cognitive judgments of life satisfaction.

### MRI Data Acquisition

The participants underwent MRI scanning on a Philips Achieva 3 Tesla (T) scanner (Philips Medical Systems, Netherlands) at the Advanced Imaging Research Center. In each imaging session, one or two 416-s runs of rsfMRI were acquired using a standard 32-channel head coil with T_2_^*^-weighted image sequence [repetition time (TR)/echo time(TE)=2000/30 ms; flip angle (FA)=80°; field of view (FOV)=22.0×22.0 cm; matrix=64×64; 37 slices, 4.0 mm thick]. Total number of rsfMRI runs was different across the participants because, at the early stage of our study, we observed that the QA procedures with only one rsfMRI run yielded high rates of participant exclusion. Thus, we additionally acquired two rsfMRI runs for the remainder of the data collection (our strategy to account for differences in total number of rsfMRI scans across the participants is detailed below). During rsfMRI acquisition, the participants were asked to remain still with their eyes closed. For rsfMRI alignment, we obtained one high resolution T_1_-weighted image of the whole brain (TR/TE=8.2/3.8 ms; FA=12°; FOV=25.6×25.6 cm; matrix=256×256; 160 slices, 1.0 mm thick) for each participant, using the same head coil.

### MRI Preprocessing

RsfMRI data were preprocessed with standard methods using a modified version of a shell script generated by afni_proc.py (http://afni.nimh.nih.gov/pub/dist/doc/program_help/afni_proc.py.html) from AFNI (Cox, 1996). Each subject’s whole-brain structural images were first skull-stripped and registered to the Montreal Neurological Institute (MNI; Evans et al., 1993) space. For each rsfMRI run, the initial four time points were discarded to allow T_1_ magnetization saturation. Standard preprocessing methods were then applied, including despiking, slice timing correction, motion correction, coregistration to the structural images in the MNI space using a single affine transform with spatial resampling (4 mm isotropic), normalization to whole brain mode of 1000, band-pass filtering (0.009< f <0.08 Hz), and linear regression.

At the motion correction stage, the six rigid body motion profiles were obtained for the linear regression. In the linear regression, the rsfMRI time-series were 3^rd^ order detrended, and several sources of signal fluctuation unlikely to be of neuronal origin were regressed out: (1) six parameters for the rigid body head motion acquired from the motion correction (Johnstone et al., 2006), (2) the signal averaged over the lateral ventricles, (3) the signal averaged over a region centered in the deep cerebral white matter, (4) the signal averaged over the whole brain [Fox et al., 2005; see the control analyses and their results for the effects of global signal regression (GSR) on network analysis results], and (5) the first temporal derivatives of aforementioned parameters.

After the linear regression, motion “scrubbing” (Power, Barnes, Snyder, Schlaggar, & Petersen, 2012) was performed with a framewise displacement (FD) of 0.5 mm and a standardized DVARS (http://www2.warwick.ac.uk/fac/sci/statistics/staff/academic-research/nichols/scripts/fsl/DVARS.sh) of 1.8 to prevent potential motion artifacts (Power et al., 2012; Satterthwaite et al., 2012; van Dijk, Sabuncu, & Buckner, 2012). A standardized DVARS of 1.8 corresponds to the median plus 1.5 times interquartile range of the standardized DVARS data across all frames and runs. The remaining rsfMRI signals were spatially blurred with 6 mm full-width-at-half-maximum (FWHM) Gaussian kernel. If two rsfMRI runs were acquired, the two preprocessed rsfMRI runs were temporally concatenated. To account for the differences in total number of frames after motion scrubbing (and different number of rsfMRI runs) across the participants and to prevent bias in estimating correlation coefficients from different degrees of freedom across rsfMRI scans, all remaining frames were trimmed to the minimum length (121 frames; 242s) across all concatenated rsfMRI scans after scrubbing as suggested in Power et al. (2014).

### Quality Assurance

We visually inspected structural MRI scans to ensure that subjects had no apparent brain atrophy. In rsfMRI preprocessing, the quality of preprocessed data was visually inspected at each step. After motion “scrubbing,” we confirmed that the total length of remaining frames after the “scrubbing” was longer than 4 min, the minimum length required to reliably estimate functional connectivity (van Dijk et al., 2010).

### Network Analyses

#### Identification of disrupted connections

To obtain a connectivity matrix for each of the subjects, we first defined nodes as 43 regions (6 mm spheres; Table 2) affiliated with the DMN, DAN, and FPCN (Spreng et al., 2013). We then calculated the Pearson correlation coefficients for time-series from each pair of the nodes. Correlation coefficients between time-series at short-distance nodes (20 mm in Euclidean distance), presumably associated with non-biological origins such as increased correlation by preprocessing and subject motion, were adjusted to be zeros (Power et al., 2011). After the Fisher’s *Z*-transform to ensure the normality of correlations, we performed the general linear model (GLM) analysis with covariates of within-group-centered BDI-II scores on each of the connectivity matrix elements due to statistically significant group differences in BDI-II scores (Table 1). Note that PCL-S scores for the TBI group were not incorporated into the GLM analysis since BDI-II and PCL-S scores for the TBI group were highly correlated (*p*<10^−4^), introducing co-linearity into the design matrix.

Statistically significant group differences in connectivity were identified at *|Z|*>2.58 (*p*<.01 at the connection level) with correction for multiple comparisons at *p*<.05 using network-based statistic (NBS; Zalesky, Fornito, & Bullmore, 2010; https://sites.google.com/site/bctnet/comparison/nbs). A total of 10,000 permutations were generated to estimate the null distribution of maximal component size. NBS-corrected group differences in connectivity were visualized on anatomical space using BrainNet Viewer (Xia, Wang, & He, 2013). The number of connections with statistically significant group differences was then identified according to (1) Euclidean distance between regions, (2) intra- *versus* inter-hemispheric connections, and (3) within- *versus* between-network connections.

### Graph theoretic analyses

#### Network construction

Weighted and undirected networks were constructed for graph theoretic analyses. We defined an edge as the Pearson correlation coefficient for time-series from a pair of the nodes. The correlation coefficients controlled for the BDI-II scores by within-group-centered BDI-II score covariates were then thresholded by the network cost, *K*, an average of suparthreshold correlation coefficients. Thresholding based on *K* allowed us to account for the potential effects of group differences in overall functional connectivity strengths on group comparisons of the subsequent network measures 0≤*K*≤1. When a correlation coefficient for thresholding increases, the network becomes more sparse, yielding low cost *K*. When a correlation coefficient for thresholding decreases, the network becomes denser, yielding high cost *K*.

As numerical values and topological properties of network measures vary with these threshold levels, we performed group analyses of the network measures as a function of *K*, starting from 0.01 in step size of 0.01. Note that, in this thresholding procedure, only positive correlation coefficients were considered for the network construction since the meaning of negative correlations in rsfMRI is unclear at the present time (see the Discussion section for relevant limitations). For each subject, networks with higher *K* were obtained until the smallest positive correlation coefficient was included. Since the maximum *K* that allows only positive correlation coefficients was different across subjects, the available number of subjects for group comparisons of the network measures decreased as we applied higher *K* (i.e., lower correlation coefficient for thresholding) across subjects. Thus, we limited the ranges of *K* for group analyses of the network measures up to *K=*0.23 at which we reliably performed group analyses (*N*≥5 per group).

#### Network measures

With the connection weight matrices, we obtained the global efficiency (Latora & Marchiori, 2001), local efficiency (Latora & Marchiori, 2001), and cost efficiency (Achard & Bullmore, 2007) for whole-brain network measures. Subsequently, we also obtained regional global efficiency and local efficiency for regional network measures. To obtain these efficiency measures, the network distance (or the shortest path length) should first be calculated. The distance between nodes *i* and *j*, *d*_*ij*_, was defined:
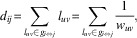
where *l*_*uv*_ is connection length between nodes *u* and 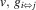 is the shortest path between nodes *i* and *j* and *w*_*uv*_ is a network weight between nodes *u* and *v*. If nodes *i* and *j* are disconnected, 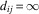.

Global efficiency, *E*_*glob*_, was defined:
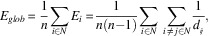
where *E*_*i*_ is regional global efficiency of node *i*. *E*_*glob*_ is a measure of how tightly, on average, nodes are connected with less indirect paths between nodes over the entire network. In theory, 0 ≤ *E*_*glob*_ ≤ 1. *E*_*glob*_ *=*0 when all nodes are disconnected. *E*_*glob*_ *=*1 when all nodes are directly connected with maximum weights (Latora & Marchiori, 2001).

Local efficiency, *E*_*loc*_, was defined:
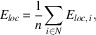
where *E*_*loc*,*i*_ is the efficiency of the neighborhood subgraph of node *i*, *N*_*i*_. Note that since the regional local efficiency, *E*_*loc*,*i*_, quantifies how much the neighbors of *i* are fault tolerant when *i* is removed (Latora & Marchiori, 2001). Cost efficiency, *E*_*cost*_, is defined as *E*_*cost*_ *=E*_*glob*_-*K*. Cost efficiency quantifies how a network is economically wired relative to the given network cost. If the network is economical, then *E*_*cost*_>0 (Achard & Bullmore, 2007). We performed all of the graph theoretic analyses using the brain connectivity toolbox in MATLAB (Rubinov & Sporns, 2010; http://www.brain-connectivity-toolbox.net/).

### Statistical Analyses

All statistical analyses were assessed in MATLAB R2013a. First, we performed the Shapiro-Wilk test at *α=*0.05 to assess the normality of distributions of each group’s demographics (age, years of education, BDI-II scores, PCL-S scores, percentage of motion-censored volumes, and average FD after motion censoring and trimming) and each network measure. Age did not pass the Shapiro-Wilk normality test. Thus, the Mann-Whitney *U* test was used to compare age between the groups. Two sample *t* tests were used to compare other demographics between the groups. The Fisher’s exact test was used to compare the gender distributions between the groups. Group comparisons on the neuropsychological test scores were carried out using the GLM with age, years of education, and within-group-centered BDI-II score covariates. Within-group-centered BDI-II score covariates were included to assess group differences in neuropsychological measures at average BDI-II scores of each respective group (Table 1).

All network measures for the control group passed the Shapiro-Wilk normality test, but most of the measures for the TBI group did not. Thus, for group comparison of the network measures, the Mann-Whitney *U* test was used.

### Control Analyses

We assessed whether there were (1) the effects of demographics (motion, age, PTSD symptom severity, post-injury time) on our results, (2) “overall” neuropsychological deficits in the TBI group, (3) effects of estimated initial injury severity, (4) effects of depressive symptoms in the TBI group, (5) effects of a mixture of civilians and veterans in the TBI group, (6) group differences in whole-brain volumes, (7) sustained DAI in the TBI group, (8) effects of an initial threshold level on NBS, (9) discrepancies in our results when using partial-correlations, (10) effects of GSR, and (11) effects of short distance nodes (see the Supplementary Material for details of the control analyses).

## Results

### Demographics Comparisons between the Groups

The TBI participants were in the long-term chronic phase of TBI (approximately 8 years post-injury time on average). There were no statistically significant differences in age, education, or gender between the groups (Table 1). There was a trend in which the TBI group participants were older than the controls. The trend in age differences between the groups was not associated with group differences in the network measures (See control analysis results). The *estimated* initial injury severity of the TBI participants was primarily mild, but the types of injury were diverse. Although the TBI participants were not *clinically* diagnosed with depression or PTSD (per inclusion criteria), the TBI group was higher on depressive symptom severity than the controls (*p*<.05). The TBI group had mild depressive symptoms on average, as measured by the BDI-II manual (Beck et al., 1996).

The average PCL-S score of the TBI group fell within the borderline range of suggested cut-off scores for PTSD screening in specialized medical clinics according to the U.S. Department of Veterans Affairs guidelines for PCL usage (http://www.ptsd.va.gov/professional/assessment/adult-sr/ptsd-checklist.asp). The presence of depressive and PTSD-related symptoms of these TBI participants was not surprising as comorbid psychiatric disorders or symptoms are common in chronic TBI (e.g., Zgaljardic et al., 2015, for review). Thus, we included within-group-centered BDI-II score covariates in all subsequent group analyses, and assessed whether the presence of comorbid depressive symptoms in the TBI group systematically altered our findings with the full TBI samples (see the Control Analysis Results and Limitations and Future Research sections).

### Neuropsychological Measures

There were no statistically significant group differences in estimated current IQ between the groups (Table 3). The TBI group did not show statistically significant differences between premorbid and current IQ. The TBI group showed significantly lower performance (*p*<.05) on the word reading condition of the color-word interference test and on the satisfaction with life scale. More TBI individuals than expected by chance (*N*=1; 2.5%) showed deficits in matrix reasoning, digit span forward and backward, all sub-domains of color-word interference, card sorting, and trail making, category switching (total correct) of verbal fluency, delayed recall, and satisfaction with life scale.

### Network-Based Statistics Results

**Fig. 1:**
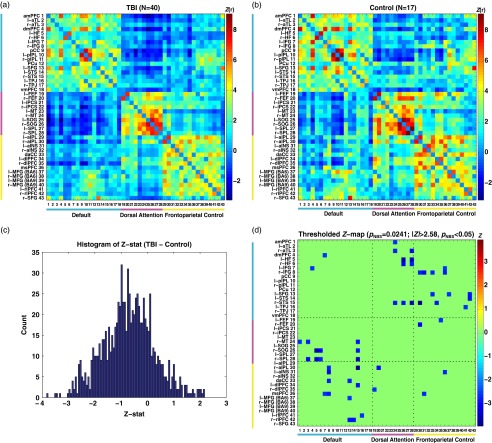
Group comparisons of average connectivity matrices. (a) Average connectivity of the TBI group. (b) Average connectivity of the control group. (c) Histogram for *Z*-statistics of group comparisons on average connectivity. (d) Thresholded Z-statistic map for group comparisons (*p*_NBS_<.05 at |*Z|*>2.58). Colorbars in (a) and (b) represent Fisher’s *Z*-transformed correlation coefficients. See Table 2 for abbreviations for the name of regions.

**Fig. 2:**
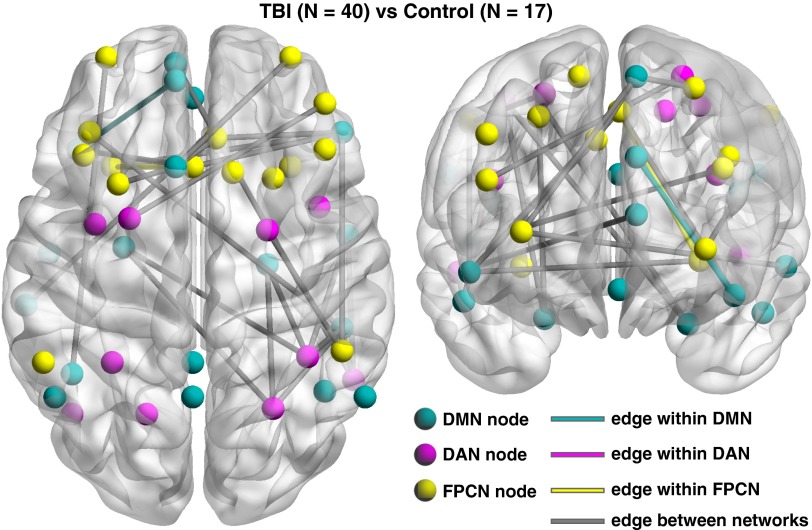
An anatomical view (dorsal and coronal view) of reduced connectivity in TBI relative to the controls (*p*_NBS_<.05 at |*Z|*>2.58). The left side is the left hemisphere.

**Fig. 3:**
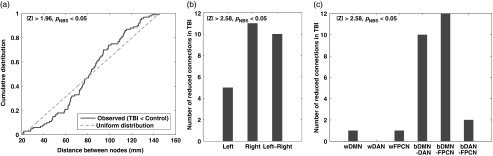
The number of reduced connections in TBI relative to the controls by (a) distance between nodes, (b) intra- *versus* inter-hemisphere, and (c) within- *versus* between-network. Note that the cumulative distribution (a) was obtained from thresholded *Z*-statistic map for group comparisons (*p*_NBS_<.05 at |*Z*|>1.96) since the total number of relatively reduced connections in TBI at |*Z*|>2.58, *p*_NBS_<.05 was small (*N*=26) to reliably estimate the cumulative distribution.

Consistent with the findings of Spreng et al. (2013), group-averaged connectivity matrices demonstrated a dissociable structure of DMN-DAN-FPCN (Figure 1A, B). Group comparisons of connectivity matrices revealed that the TBI group exhibited reductions in connectivity among the three networks compared to controls (Figure 1C, D). An anatomical view of reduced connectivity within the TBI group relative to the controls (Figure 2) highlights the fact that disruptions occurred more frequently in connections between the three networks compared to those within the networks. Furthermore, the cumulative distribution of the number of reduced connections in TBI over controls skewed toward long-range connections (Figure 3A), and notable disruptions occurred in inter-hemispheric connections (Figure 3B). Among the three types of between-network connections, DMN-FPCN and DMN-DAN were prominently disrupted (Figure 3C).

### Whole-Brain Network Properties

**Fig. 4:**
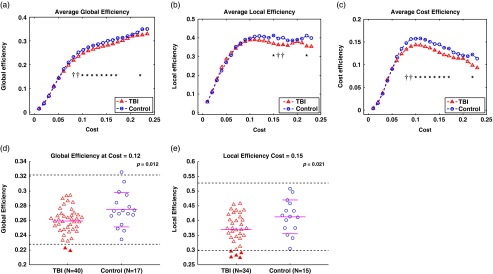
Global, local and cost efficiency of the TBI group and the controls. (a–c) Average global, local, and cost efficiency as a function of network cost, respectively. Note that, to reliably perform group analyses, we limited ranges of network cost (from 0.01 to 0.23 in step size of 0.01) to include N≥5 per group. * and † represent *p*<.05 and *p*<.1, respectively, at the given network cost level. (d,e) Scatter plots for global and local efficiency at network costs of 0.12 and 0.15, respectively. The I bars indicate the means and standard deviation of the controls, the dotted horizontal bar is two standard deviations from the mean of the controls and the solid horizontal bar in the TBI group is the mean of the TBI group. Filled triangles represents TBI individuals with relatively “abnormal” efficiency, located outside the dotted horizontal bars. The *p*-values were obtained from the Mann-Whitney *U* test.

Overall, the TBI group showed relative reductions in efficiency measures at the whole-brain level (Figure 4). The TBI group showed statistically significant reductions in (1) global and cost efficiency occurring for 0.1 ≤ *K* ≤ 0.17 and *K=*0.22 at *p*<.05 and *K=*0.08, 0.09 at *p*<.1 and (2) local efficiency for *K=*0.15, 0.22 at *p*<.05 and *K=*0.16, 0.17 at *p*<.1 (Figure 4A–C). Importantly, reductions in global and local efficiency occurred when the network was highly economical. Scatter plots for global and local efficiency at *K=*0.12 and 0.15, respectively, (Figure 4D–E) revealed relative reductions of the TBI group in these measures at the single-subject level and TBI individuals with “abnormally” low global and local efficiency. Taken together, notable reductions in global and local efficiency over high network cost in the TBI group indicate the detrimental effects of TBI-related disruptions in weak but important long-range, inter-hemispheric, and between-network connectivity (Figures 1–4).

### Regional Network Properties

**Fig. 5:**
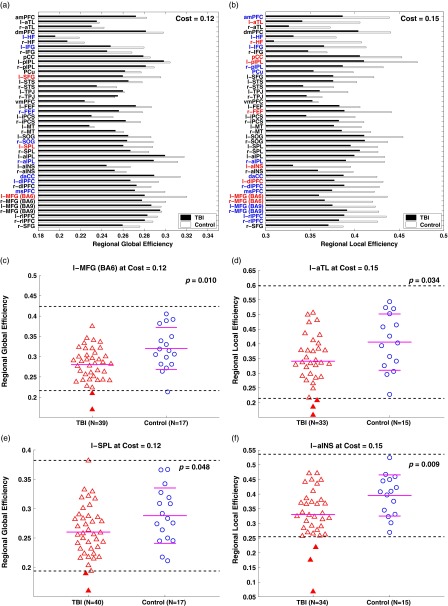
Regional, global, and efficiency of the TBI group and the controls at network costs of 0.12 and 0.15, respectively. (a,b) Bar graphs for average regional global and local efficiency, respectively. Red and blue colors represent brain regions with *p*<.05 and *p*<.1, respectively. (c,e) Scatter plots for regional global efficiency of the selected regions. (d,f) Scatter plots for regional local efficiency of the selected regions. See Table 2 for abbreviations and Figure 4 for the details of the scatter plots.

Assessments of the regional global and local efficiency for *K=*0.12 and 0.15, respectively, exhibited relative reductions in the global and local efficiency in the TBI group at each of the regions (Figure 5A, B). Of the two efficiency measures, the TBI group showed more prominent reductions in regional local efficiency than in regional global efficiency. Specifically, statistically significant group differences in regional global efficiency (*p*<.05) occurred in three regions [l-SFG, l-SPL, and l-MFG (BA6)] and noticeable group differences in the regional global efficiency (*p*<.1) occurred in eight regions (l-HF, l-IFG, r-FEF, r-SOG, r-aIPL, daCC, l-dlFPC, and msPFC). As for the regional local efficiency, statistically significant group differences (*p*<.05) occurred in 9 regions [l-aTL, r-HF, pCC, l-pIPL, r-FEF, l-aINS, l-dlPFC, l-MFG (BA6), and r-MFG (BA6)] and noticeable group differences (*p*<.1) occurred in other 13 regions (amPFC, l-HF, l-IFG, r-pIPL, PCu, r-aIPL, daCC, r-dlPFC, msPFC, l-MFG (BA 9), r-MFG (BA9), l-rlPFC, and r-rlPFC).

### Control Analysis Results

#### Effects of demographics

Our motion analysis (Table 1) revealed that there were no statistically significant group differences in the percentage of motion-censored volumes or average FD after motion censoring and trimming. The number of connections that were correlated with age at α=0.05 was less than 5%, indicating minimal effects of age on the connectivity matrices in our groups. The global and local efficiency were not associated with the amount of motion (Supporting Figure S1) or age (Supporting Figure S2A, D) in any of the groups. PCL-S scores and post-injury times did not show an influence on the global or local efficiency (Supporting Figure S2B–C, E–F).

#### Neuropsychological deficits in the TBI group

More TBI participants than would be expected by chance (12/40 *vs*. 1/40 expected, *p=*.0007) had abnormally poor neuropsychological performance relative to the controls in more than two neuropsychological measures (Figure S3A), indicating overall deficits in neuropsychological measures for the TBI group. Group comparisons of global and local efficiency in the TBI sub-group with neuropsychological deficits (*N*=26) *versus* the TBI sub-group without any neuropsychological deficits (*N*=14) did not show statistically significant group differences (Figure S3B).

#### Effects of estimated initial injury severity

There were no systematic effects of estimated *initial* injury severity on the TBI participants’ BDI-II or PCL-S scores, six selected neuropsychological measures, or global or local efficiency (Supporting Figure S4). There were also no systematic effects of estimated initial injury severity on our NBS findings (Supporting Figure S5; see Supplementary Materials for details).

#### Effect of depressive symptoms

We found no systematic effects of comorbid depressive symptoms on our findings on NBS (Supporting Figure S6) or network efficiency measures (see Supplementary Materials for details).

#### Effects of a mixture of civilians and veterans with TBI

There were no systematic effects of military status (civilian or veteran with TBI) on our NBS findings (Supporting Figure S7) or network efficiency measures (see Supplementary Materials for details).

#### Whole-brain volumes

There were no statistically significant group differences in whole-brain volumes (Table 1).

#### Sustained DAIs

The TBI group had relatively reduced fractional anisotropy in the anterior thalamic radiation, corpus callosum, corticospinal tract, forceps major and minor, inferior fronto-occipital fasciculus, and superior and inferior longitudinal fasciculi, confirming sustained DAIs among the TBI individuals (Supporting Figure S8).

#### Effects of an initial threshold level on NBS

NBS analyses with two additional threshold levels (Supporting Figure S9) replicated the patterns of relatively reduced connectivity of the TBI group in Figure 2.

#### Partial correlation

Consistent with the full correlation analysis results, group comparisons of direct connectivity using partial correlation coefficients revealed relative reductions in connectivity for the TBI group (Supporting Figure S10). There were a few exceptions showing relatively increased connectivity (Supporting Figure S10). Again, NBS analyses with partial correlations allowed us to demonstrate disrupted patterns of the TBI group (Supporting Figure S11) consistent with the patterns in Figure 2.

#### Effects of global signal regression

Analyses of the global and local efficiency without GSR (Supporting Figure S12) essentially replicated the results after GSR in Figure 4 with a broader range of *K*.

#### Effects of short distance nodes

Retaining connectivity strengths between short distance nodes did not markedly alter disruption patterns of long-range connections (Supporting Figure S13).

## Discussion

We characterized disrupted resting-state functional connectivity of DMN-DAN-FPCN in chronic TBI comparing to healthy individuals. We confirmed that there were disruptions in chronic TBI individuals not only within-network connectivity but also between-network connectivity (Figures 1–3). Of the three types of between-network connectivity, DMN-FPCN and DMN-DAN showed marked reductions in the TBI group (Figures 2–3). Furthermore, quantitative analysis revealed that the patterns of disruptions occurred predominantly in long-range and inter-hemispheric connections in the TBI group (Figure 3). Lastly, graph theoretic analyses demonstrated reduced efficiency in the TBI group over the controls at both whole-brain (Figure 4) and regional levels (Figure 5).

### Advantages of Combining both Complex Network and Hypothesis-Driven Approaches to Identify Disrupted Networks in TBI

This study demonstrates the advantages of combining both complex network and hypothesis-driven approaches over either simple network or whole-brain-wide data-driven approaches only in identifying network disruptions in chronic TBI relevant to specific deficits. As such, comprehensive assessments of multiple networks-of-interest allowed us to identify markedly disrupted between-network connectivity of chronic TBI individuals (Figures 1–3). These pronounced disruptions in between-network connectivity in chronic TBI would have been missed if we had simply investigated the integrity of a single network or connectivity of a single-brain region with rest of the brain.

On the other hand, data-driven complex network approaches for TBI individuals at the whole-brain level (e.g., Han et al., 2014; Nakamura et al., 2009) present challenges in interpreting behavioral consequences from the identified network disruptions. Our study translated the advantages of hypothesis-driven simple network approaches (i.e., more straightforward interpretations of the behavioral consequences of network disruptions) into complex network analysis by constraining the networks-of-interest in the context of goal-directed cognition. Combining the advantages of both approaches, we demonstrated that disrupted interactions of DMN-DAN-FPCN may be a potential neural mechanism underlying the deficits in goal-directed cognition observed in chronic TBI individuals. Note that this study does not provide direct evidence to support this hypothesis, thus further studies are needed to test it (see the Limitations and Future Research section). Given the demonstrated advantages of investigating multiple networks specific to goal-directed cognition, we encourage others to assess interactions among multiple networks-of-interest to better understand deficits in higher order cognitive functions such as reasoning, decision making, and selective attention in TBI and in other disorders involving white matter injuries or degeneration.

### Findings Relative to Previous Studies

#### Reduced long-range connectivity of DMN-DAN-FPCN in chronic TBI

Our study demonstrated predominantly reduced strengths in long-range connectivity in the TBI group (Figures 2, 3A). This finding is consistent with the disruption-patterns reported in other functional connectivity studies in TBI (Castellanos et al., 2010; Kumar, Rao, Chandramouli, & Pillai, 2009). Diffusion tensor imaging (DTI) studies in TBI also revealed frequent abnormalities in long-range white matter tracts such as the superior and inferior longitudinal fasciculi, inferior fronto-occipital fasciculus (Messe et al., 2011; Niogi et al., 2008; Singh, Jeong, Hwang, Sungkarat, & Gruen, 2010; Smits et al., 2011), cingulum bundles (Mac Donald et al., 2011; Niogi et al., 2008), and fornix (Singh et al., 2010). Thus, observed disruptions in long-range functional connectivity among the three networks in chronic TBI may be attributed to abnormalities in long-range white matter tracts mediating multiple networks. However, future studies directly comparing structural and functional connectivity will be required to confirm this possibility as relationships between functional and structural connectivity may not be straightforward (Honey et al., 2009).

#### Reduced inter-hemispheric connectivity of DMN-DAN-FPCN in chronic TBI

Consistent with the reported whole-brain-wide reductions in inter-hemispheric functional connectivity of TBI individuals in previous studies (Kumar et al., 2009; Marquez de la Plata et al., 2011; Rigon, Duff, McAuley, Kramer, & Voss, 2015; Slobounov et al., 2011; Sours et al., 2015), the TBI group showed relative reductions in inter-hemispheric connectivity of DMN-DAN-FPCN (Figures 2, 3B). Such reductions in inter-hemispheric functional connectivity may be attributable to damage to the corpus callosum, which is common in TBI (Niogi & Mukherjee, 2010). Reductions in inter-hemispheric functional connectivity occur after corpus callosum resection in rhesus monkeys (O’Reilly et al., 2013). However, further studies directly evaluating the effects of corpus callosum damage on connectivity in humans will be required to confirm this speculation. Nonetheless, our findings of reduced inter-hemispheric functional connectivity in chronic TBI indicate that damage to regions in one hemisphere impact intact regions in the other hemisphere, supporting the use of network approaches for chronic TBI individuals.

#### Disruptions in between-network connectivity of DMN-DAN-FPCN in chronic TBI

Marked reductions in between-network connectivity over within-network connectivity in our TBI group (Figures 1D, 2, 3C) are consistent with the disruption patterns reported in Han et al. (2014) whereby veterans with sub-acute, concussive blast-related TBI showed marked reductions in between-module connectivity with minimal alterations in within-module connectivity. We extended efforts to identify altered between-network interactions following TBI. Bonnelle et al. (2012) demonstrated that the amount of white matter damage in the SN predicts the abnormality of the DMN in chronic TBI. In our study, we further revealed disrupted between-network interactions in TBI by *directly* measuring functional connectivity among the three networks.

Although our study was not able to directly show which cognitive deficits were associated with reduced DMN-FPCN connectivity in the TBI group, potentially the most relevant deficits may be autobiographical planning and future problem solving. Several studies (Baird, Smallwood, & Schooler, 2011; Gerlach, Spreng, Gilmore, & Schacter, 2011; Gerlach, Spreng, Madore, & Schacter, 2014; Spreng et al., 2010) demonstrated that autobiographical planning and future problem solving tasks induce activity in the regions within both DMN and FPCN, presumably by engaging the autobiographical memory system and executive control process (Smallwood, Brown, Baird, & Schooler, 2012). In TBI, deficits in autobiographical content and their associations with everyday planning difficulties have also been reported (Dritschel, Kogan, Burton, Burton, & Goddard, 1998).

Of interest, relative reductions in DMN-DAN connectivity in the TBI group occurred in connections where the controls retained positive connectivity (e.g., connections of the DAN with r-aTL, l-HF, and r-STS in the DMN). Prior literature indicated an antagonistic relationship in DMN-DAN, supported by observed anti-correlation between the DMN and DAN in Fox et al. (2005) and Kelly, Uddin, Biswal, Castellanos, and Milham (2008). Furthermore, disrupted anti-correlation between the DMN and task-related networks in TBI were demonstrated (Mayer et al., 2011; Sours et al., 2013). Thus, relative reductions in positive DMN-DAN connectivity of the TBI group apparently contradicts previous findings of negative DMN-DAN connectivity (Fox et al., 2005; Kelly et al., 2008). However, Dixon, Fox, and Christoff (2014) proposed that activation within the DMN and DAN could co-occur with minimal interference if externally or internally directed cognition involves spontaneous processing such as in creative thinking (Ellamil, Dobson, Beeman, & Christoff, 2012) and the influence of self-evaluative thoughts on cognitive control (Bengtsson, Dolan, & Passingham, 2011).

#### Reduced connectivity versus elevated connectivity in TBI

We observed markedly reduced functional connectivity in the TBI individuals relative to the control participants; however, there is a body of literature that has reported elevated functional connectivity in TBI (Caeyenberghs et al., 2012, 2013; Hillary et al., 2014; Nakamura et al., 2009; Sharp et al., 2011). Such discrepant reports in the literature may be attributable to several factors that differed across studies. First, Caeyenberghs et al. (2012, 2013) and Hillary et al. (2014) reported their findings based on task-state functional connectivity. The directionality of task-state functional connectivity in our TBI group has not been established (see the Limitations and Future Research section in this regard).

Second, Caeyenberghs et al. (2012, 2013) and Hillary et al. (2014) obtained partial-correlations, while we obtained both full- and partial-correlations. Although our TBI group showed relatively reduced full-correlation coefficients, relative increases in connectivity were also observed over some connections with the partial correlation analysis (Supporting Figures S10, S11).

Third, the directionality of abnormal connectivity in individuals with TBI can vary with seed regions, networks-of-interests, and different definition of nodes [e.g., within- *vs*. between-module connectivity in Han et al. (2014)], and these parameters in our study were different than the other studies that have reported elevated connectivity (Caeyenberghs et al., 2012, 2013; Hillary et al., 2014; Nakamura et al., 2009).

Fourth, differences in sample characteristics could play a role in the directionality of abnormal functional connectivity in TBI. As such, Hillary et al. (2014) and Nakamura et al. (2009) assessed TBI individuals at 3 and 6 months post-injury, whereas our TBI individuals were sampled at several years post-injury.

Fifth, to assess connectivity in TBI, Sharp et al. (2011) performed ICA and dual regression, while we performed the Pearson correlation. Note that the ICA-plus-dual-regression and Pearson correlation-based approaches are conceptually different (e.g., Joel, Caffo, van Zijl, & Pekar, 2011). The ICA-plus-dual-regression approach decomposes resting-state fMRI signal at a voxel according to the each of the networks (i.e., the within-network component only). Thus, the ICA-plus-dual-regression approach does not take account the between-network component of connectivity at the voxel-level (although ICA can yield between-network connectivity at the network-level using network-level time-courses).

Indeed, the same research group responsible for the study of Sharp et al. (2011) used the Pearson correlation which takes account for between-network components at the region-level and they have demonstrated overall reductions in resting-state functional connectivity in individuals with TBI (Pandit et al., 2013). Furthermore, the dual-regression controls for the time-courses of networks other than the given network-of-interest. This is conceptually similar to the partial correlation approach that controls for correlations of time-courses at other regions outside a pair of regions-of-interest. Note that, when we used the partial correlation approach, we observed relatively elevated connectivity for our TBI group primarily in within-network connections. Taken together, mixed reports on reduced and elevated connectivity in TBI literature demonstrate complex nature of TBI and a strong need for additional research across methods and populations with TBI. We did evaluate the potential effects of depressive symptoms and PTSD scores for TBI participants on our results, but did not find substantive differences in network connectivity attributable to these factors.

#### Reduced efficiencies of DMN-DAN-FPCN in chronic TBI

Graph theoretic analyses revealed the consequences of impaired long-range, inter-hemispheric and between-network connectivity of DMN-DAN-FPCN in chronic TBI (Figures 4, 5). In healthy subjects, functional brain networks are “economical” in that the brain exhibits high global and local efficiency of parallel information processing given low wiring cost (Achard & Bullmore, 2007). In our TBI group, network disruptions led to individuals circumventing impaired weak-but-efficient long-range, inter-hemispheric and between-network connections by using less efficient and “noisier” alternative paths for communications. Such selections of alternative paths in DMN-DAN-FPCN implicates DMN-DAN-FPCN connectivity of the TBI group were inefficient for parallel information flow and less tolerant to additional injuries to brain regions, demonstrated by relatively low global and local efficiency, respectively (Latora & Marchiori, 2001).

Our findings of reduced global and local efficiency in chronic TBI are consistent with a previous finding on reduced efficiency in chronic TBI (Pandit et al., 2013). However, our findings are inconsistent with the study results of Nakamura et al. (2009) demonstrating no group differences in efficiency measures at the chronic stage. Such inconsistencies may be explained by the different group characteristics (*N*=6 and severe TBI) and methodologies (whole-brain connectivity) in Nakamura et al. (2009). We extend upon previous studies (Nakamura et al., 2009; Pandit et al., 2013) by providing critical new information on reduced efficiency of the brain networks in TBI in that statistically significant reductions in global and local efficiency of the TBI group occurred when cost efficiency was optimal. Such findings suggest that chronic TBI individuals would have less optimal axonal wiring relative to controls or differences in metabolic running costs to provide parallel information processing among DMN-DAN-FPCN even under the individual’s most economical network settings (Achard & Bullmore, 2007).

### Limitations and Future Research

The present study has several limitations. First, our TBI group was comprised of a mixture of individuals with *probable* mild, *probable* moderate, and *probable* severe TBI, which yields it less comparable to studies of individuals in the sub-acute (3–6 months post-injury) and short-term chronic (6 months–2 years post-injury) stages of TBI. However, at the long-term chronic stage of TBI (>2 years post-injury), *initial* injury severity often plays less critical role in characterizing TBI individuals at the time of study (Arciniegas et al., 2000; Kinnunen et al., 2010) and chronic TBI studies occasionally have reported a mixture of different injury severity levels (Bonnelle et al., 2012, 2011; Ham et al., 2014; Kinnunen et al., 2010; Schönberger et al., 2011; Sharp et al., 2011). Furthermore, the fifth edition of American Psychiatric Association’s Diagnostic and Statistical Manual of Mental Disorders (DSM-V) suggests that *initial* injury severity does not necessarily correspond to the severity of resulting neuro-cognitive disorders after TBI, and that the course of recovery depends not only on the specifics of the injury but also on co-factors such as age, prior history of brain damage, and history of substance abuse.

In our case, there were no systematic effects of estimated *initial* injury severity on the BDI-II or PCL-S scores, selected neuropsychological measures, efficiency measures, or patterns of disrupted connectivity (Supporting Figures S4, S5). Nonetheless, care should be taken in interpreting our findings with regard to TBI severity as initial injury severity was retrospectively *estimated*.

Second, a portion of the TBI sample showed comorbid depressive symptoms, which are common in chronic TBI populations. Although comorbid psychiatric symptoms are common in chronic TBI and the presence of depressive symptoms did not alter our findings (Supporting Figures S6, S7), care should be taken due to (1) potential bias in self-reported BDI-II scores driven by frequent impairments in self-awareness among TBI individuals (Malec, Testa, Rush, Brown, & Moessner, 2007) and (2) relatively small sample size of the TBI sub-groups.

Third, the behavioral consequences of the phenomena reported in this study are as yet unclear. To identify behavioral consequences of disrupted between-network connectivity in chronic TBI, task-based fMRI brain activation studies are of interest.

Fourth, it is unknown that how disrupted resting-state functional connectivity among DMN-DAN-FPCN in TBI could influence “reconfiguration” of functional connectivity in specific tasks.

Fifth, we excluded negative correlations in our network analyses due to ongoing debates about the meaning of negative correlations after GSR (Anderson et al., 2011; Chai, Castanon, Ongur, & Whitfield-Gabrieli, 2012; Chang & Glover, 2009; Fox, Zhang, Snyder, & Raichle, 2009; Murphy, Birn, Handwerker, Jones, & Bandettini, 2009; Saad et al., 2012). Note that, even though alternative approaches to reliably estimate negative correlations have been proposed (e.g., Jo, Saad, Simmons, Milbury, & Cox, 2010), the meaning of negative correlations are unclear at the present time. As such, negative correlations in rsfMRI could reflect accumulated phase delay along the path that connects two regions with positively correlated multiple edges (Chen, Chen, Xie, & Li, 2011), rather than an antagonistic relationship between two regions exhibiting a negative correlation. Assessment of anti-correlations among the three networks would be of interest if future studies clarify the meaning of negative correlations.

Our future works include an assessment of relationship between disrupted networks and behavioral consequences in this cohort and efforts to address the other concerns discussed above. Furthermore, we will identify if and how disrupted connectivity among DMN-DAN-FPCN in chronic TBI could be reorganized following rehabilitation.

In conclusion, we demonstrated pronounced disruptions in DMN-DAN-FPCN connectivity. Our findings suggest that the three networks should be analyzed together to better understand deficits in goal-directed cognition and other higher order cognitive functions in chronic TBI. Further studies are required to explain the behavioral consequences of the phenomena reported in this study.

**Table 2:** Abbreviations for the name of regions^a^

Index	Abbreviation	Region name	Index	Abbreviation	Region name
1	amPFC	Anterior medial prefontal cortex	23	r-iPCS	Right inferior precentral sulcus
2	l-aTL	Left anterior temporal lobe	24	l-MT	Left middle temporal motion complex
3	r-aTL	Right anterior temporal lobe	25	r-MT	Right middle temporal motion complex
4	dmPFC	Dorsal medial prefrontal cortex	26	l-SOG	Left superior occipital gyrus
5	l-HF	Left hippocampal formation	27	r-SOG	Right superior occipital gyrus
6	r-HF	Right hippocampal formation	28	l-SPL	Left superior parietal lobule
7	l-IFG	Left inferior frontal gyrus	29	r-SPL	Right superior parietal lobule
8	r-IFG	Right inferior frontal gyrus	30	l-aIPL	Left anterior inferior parietal lobule
9	pCC	Posterior cingulate cortex	31	r-aIPL	Right anterior inferior parietal lobule
10	l-pIPL	Left posterior inferior parietal lobule	32	l-aINS	Left anterior insula
11	r-pIPL	Right posterior inferior parietal lobule	33	r-aINS	Right anterior insula
12	PCu	Precuneus	34	daCC	Dorsal anterior cingulate cortex
13	l-SFG	Left superior frontal gyrus	35	l-dlPFC	Left dorsolateral prefrontal cortex
14	r-SFG	Right superior frontal gyrus	36	r-dlPFC	Right dorsolateral prefrontal cortex
15	l-STS	Left superior temporal sulcus	37	msPFC	Medial superior prefrontal cortex
16	r-STS	Right superior temporal sulcus	38	l-MFG (BA 6)	Left middle frontal gyrus BA 6
17	l-TPJ	Left temporal parietal junction	39	r-MFG (BA 6)	Right middle frontal gyrus BA 6
18	r-TPJ	Right temporal parietal junction	40	l-MFG (BA 9)	Left middle frontal gyrus BA 9
19	vmPFC	Ventral medial prefrontal cortex	41	r-MFG (BA 9)	Right middle frontal gyrus BA 9
20	l-FEF	Left frontal eye fields	42	l-rlPFC	Left rostrolateral prefrontal cortex
21	r-FEF	Right frontal eye fields	43	r-rlPFC	Right rostrolateral prefrontal cortex
22	l-iPCS	Left inferior precenral sulcus			

a See Spreng et al., 2013, for coordinates and network affiliations for the listed regions.

**Table 3:** Neuropsychological assessment results

Neuropsychological measure^a^	TBI (*N*=40)	Controls (*N*=17)	*p*-Values^b^	‘Abnormal’ TBI^c^
Similarities	37.3±4.4	38.1±5.7	0.48	0
Matrix reasoning	28.4±4.8	30.2±2.9	0.37	**4**
WASI FSIQ-2 (current IQ)	110.6±10.9	111.6±14.8	0.49	1
WTAR FSIQ (premorbid IQ)	110.9±8.2	N/A	0.84^d^	N/A
Digit span forward	10.4±2.4	11.0±2.6	0.38	**2**
Digit span backward	7.4±2.5	7.9±2.2	0.37	**5**
Color-word: Color naming (s)	32.0±8.6	27.2±5.7	0.06^**†**^	**8**
Color-word: Word reading (s)	23.7±6.1	20.6±4.3	0.03^*^	**7**
Color-word: Inhibition (s)	57.1±15.8	49.6±14.8	0.12	**3**
Color-word: Inhibition/switching (s)	63.9±16.7	57.0±13.9	0.12	**6**
Verbal fluency: Letter fluency, total correct	41.1±10.2	42.2±13.3	0.71	0
Verbal fluency: Category fluency, total correct	42.2±9.8	42.7±9.1	0.61	1
Verbal fluency: Category switching, total correct	14.8±2.7	14.5±2.3	0.70	**2**
Verbal fluency: Category switching, total switching accuracy	13.9±2.8	13.0±2.5	0.38	1
Sorting: Free sorting, confirmed correct sorts	9.8±2.3	10.7±2.1	0.22	**3**
Sorting: Free sorting, description score	37.9±9.7	42.5±8.3	0.12	**5**
Sorting: Sort recognition, description score	35.5±11.0	42.4± 9.3	0.10^**†**^	**6**
Sorting: Combined description score	73.3±18.8	84.9±15.5	0.08^**†**^	**7**
Trail making: Visual scanning (s)^e^	18.7±5.1	16.8±4.1	0.17	**5**
Trail making: Number sequencing (s)	28.2±10.0	24.0±7.9	0.15	**5**
Trail making: Letter switching (s)	27.9±12.7	24.9±6.7	0.59	**4**
Trail making: Number-letter switching (s)	75.8±36.2	58.9±15.0	0.08^**†**^	**8**
Trail making: Motor speed (s)	22.4±9.4	19.3±5.7	0.25	**6**
Logical memory I: Immediate recall	13.4±4.3	13.1±5.0	0.94	1
Logical memory II: Delayed recall	11.3±5.3	12.5±4.6	0.27	**4**
Verbal problem solving	12.3±1.8	12.7±2.3	0.28	0
Visual selective learning task	113.6±32.8	128.5±42.1	0.17	0
Satisfaction with life scale	18.2±7.6	27.2±4.2	<10^−3,*^	**24**

*Note*: WASI, Wechsler Abbreviated Scale of Intelligence; FSIQ, Full Scale Intelligent Quotient; WTAR, Wechsler Test of Adult Reading.

a Mean and standard deviation values were reported.

b *p*-Values were obtained with age, years of education and within-group-centered BDI-II score covariates.*, † indicate *p*<0.05, *p*<0.1, respectively.

c The number of “abnormal” TBI participants whose neuropsychological measures were outside (either above for the color-word and the trail making tests or below for the other measures) the two-standard deviation-band from the mean of the controls. Data in boldface indicate that the number of TBI participants who performed “abnormally” poor in a given neuropsychological measure was higher than the number of “abnormal” TBI participants expected to occur by chance [i.e., 1 out of 40 (2.5%)].

d WASI FSIQ-2 *versus* WTAR FSIQ within the TBI group.

e Unavailable for one healthy individual due to timer malfunction.

## Supplementary material

For supplementary material accompanying this paper visit http://dx.doi.org/10.1017/S1355617715001393. click here to view supplementary material
