# Supplementary material for: Disrupted Intrinsic Connectivity among Default, Dorsal Attention, and Frontoparietal Control Networks in Individuals with Chronic Traumatic Brain Injury
Source: J Int Neuropsychol Soc. 2016 Feb;22(2):263–79. doi: 10.1017/S1355617715001393 (PMC4763346; doi:10.1017/S1355617715001393)
Supplement: Supplementary file 1 [file S13556177150013935sup.zip › S1355617715001393sup001.pdf]

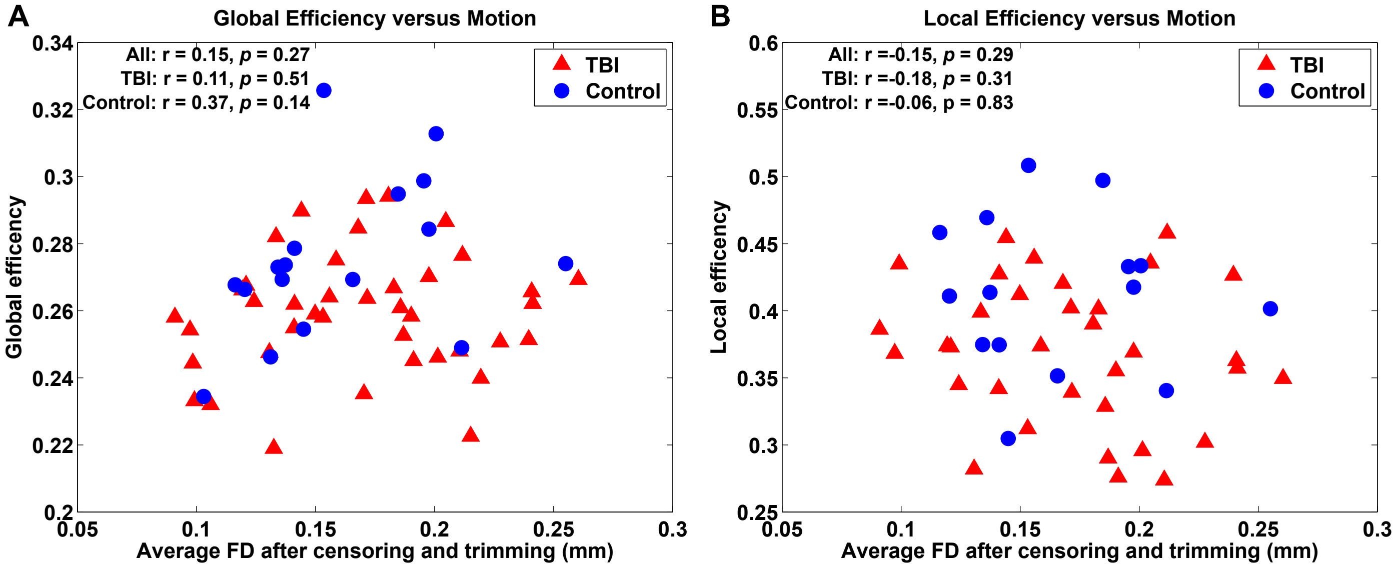

Fig. S1. Average framewise displacement (FD) of each participant after motion censoring and trimming versus the average global (A) and local (B) efficiency at network costs of 0.12 and 0.15, respectively. The r-values are the Pearson correlation coefficients.
