# Supplementary material for: Disrupted Intrinsic Connectivity among Default, Dorsal Attention, and Frontoparietal Control Networks in Individuals with Chronic Traumatic Brain Injury
Source: J Int Neuropsychol Soc. 2016 Feb;22(2):263–79. doi: 10.1017/S1355617715001393 (PMC4763346; doi:10.1017/S1355617715001393)
Supplement: Supplementary file 1 [file S13556177150013935sup.zip › S1355617715001393sup002.pdf]

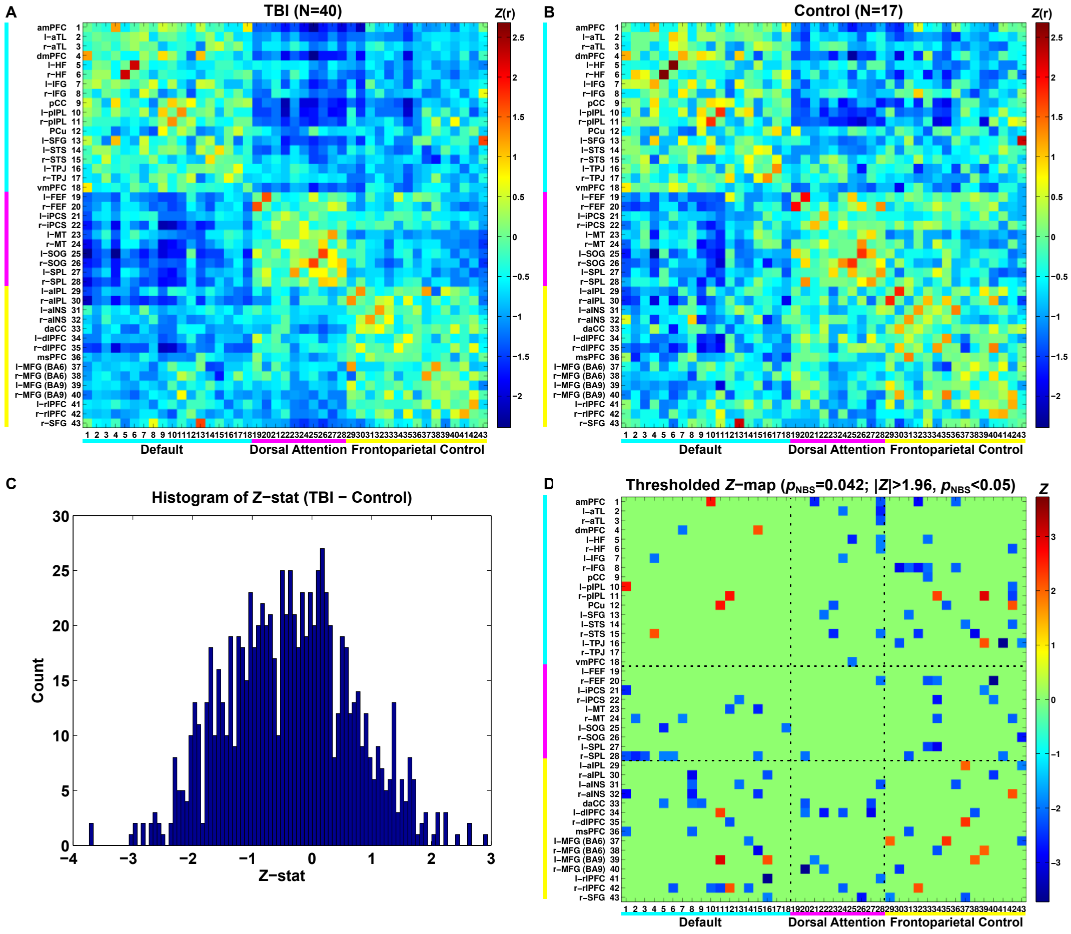

Fig. S10. Group comparisons of average connectivity matrices based on partial correlation coefficients. (A): Average connectivity of the TBI group. (B): Average connectivity of the control group. (C): Histogram for *Z*-statistics of group comparisons on average connectivity. (D) Thresholded *Z*-statistic map for group comparisons ($p_{NBS}<0.05$ at $|Z|>1.96$). Colorbars in (A) and (B) represent Fisher's *Z*-transformed correlation coefficients.
