# Supplementary material for: Disrupted Intrinsic Connectivity among Default, Dorsal Attention, and Frontoparietal Control Networks in Individuals with Chronic Traumatic Brain Injury
Source: J Int Neuropsychol Soc. 2016 Feb;22(2):263–79. doi: 10.1017/S1355617715001393 (PMC4763346; doi:10.1017/S1355617715001393)
Supplement: Supplementary file 1 [file S13556177150013935sup.zip › S1355617715001393sup003.pdf]

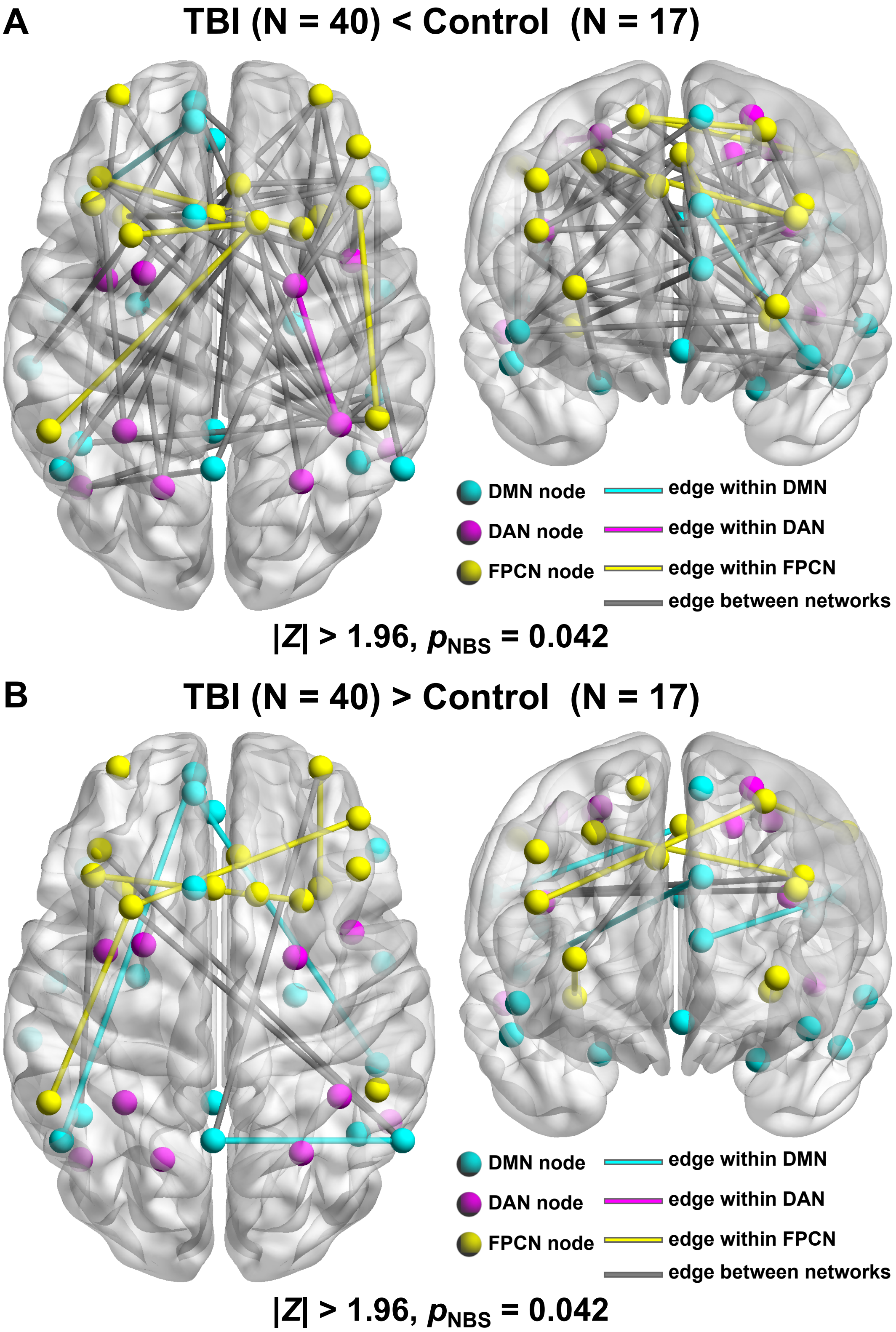

Fig. S11. An anatomical view of reduced (A) and elevated (B) connectivity in TBI relative to the controls (p_{NBS}<0.05 at |Z|>1.96) based on partial correlation coefficients. The left side is the left hemisphere.
