# Supplementary material for: Disrupted Intrinsic Connectivity among Default, Dorsal Attention, and Frontoparietal Control Networks in Individuals with Chronic Traumatic Brain Injury
Source: J Int Neuropsychol Soc. 2016 Feb;22(2):263–79. doi: 10.1017/S1355617715001393 (PMC4763346; doi:10.1017/S1355617715001393)
Supplement: Supplementary file 1 [file S13556177150013935sup.zip › S1355617715001393sup004.pdf]

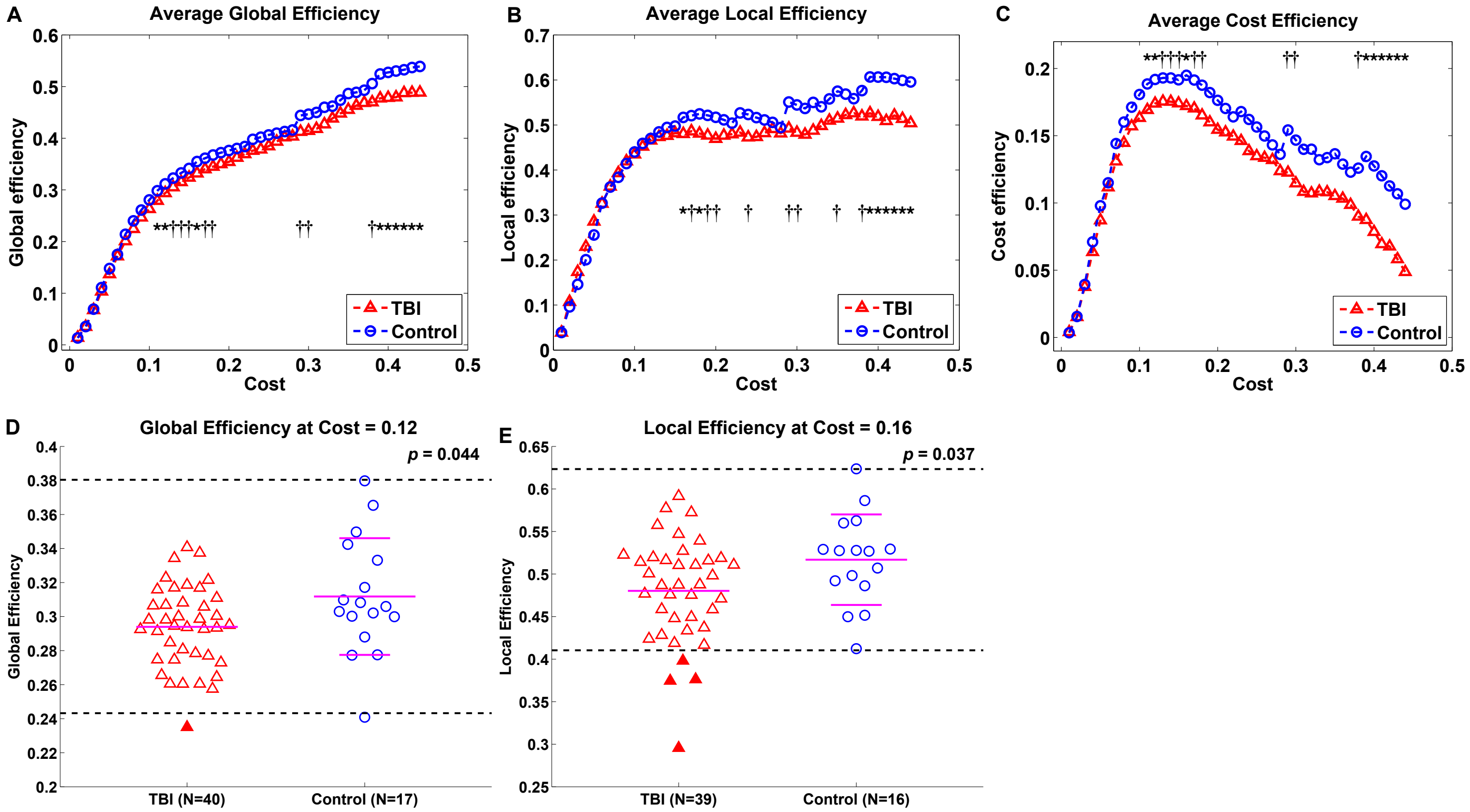

Fig. S12. Global, local and cost efficiency of the TBI group and the controls without global signal regression. (A)-(C): Average global, local and cost efficiency as a function of network cost, respectively. Note that, to reliably perform group analyses, we limited ranges of network cost (from 0.01 to 0.44 in step size of 0.01) to include N≥5 per group. (D), (E): Scatter plots for global and local efficiency at network costs of 0.12 and 0.16, respectively. See Fig. 4 for the details of the scatter plots.
