# Supplementary material for: Disrupted Intrinsic Connectivity among Default, Dorsal Attention, and Frontoparietal Control Networks in Individuals with Chronic Traumatic Brain Injury
Source: J Int Neuropsychol Soc. 2016 Feb;22(2):263–79. doi: 10.1017/S1355617715001393 (PMC4763346; doi:10.1017/S1355617715001393)
Supplement: Supplementary file 1 [file S13556177150013935sup.zip › S1355617715001393sup005.pdf]

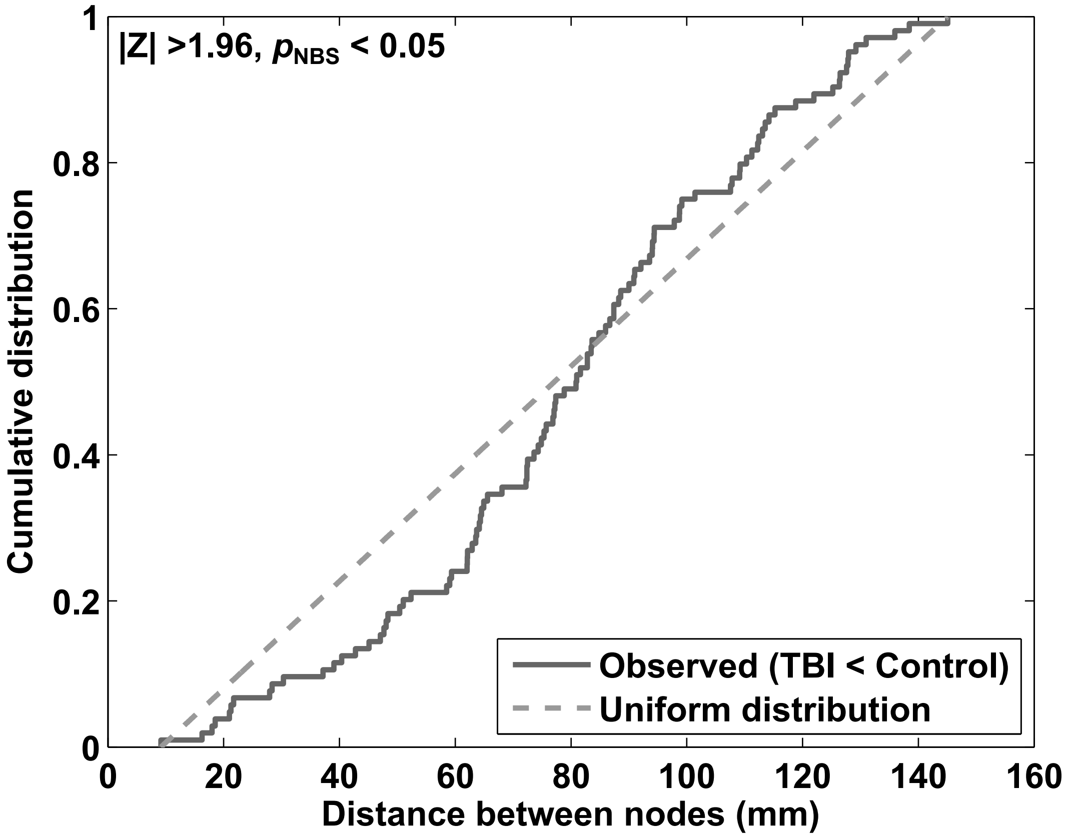

Fig. S13. The number of reduced connections in TBI relative to the controls (thresholded at $|Z| >1.96$, $p_{NBS}<0.05$) by distance between nodes while retaining connectivity strengths between short distance nodes (< 20 mm).
