# Supplementary material for: Disrupted Intrinsic Connectivity among Default, Dorsal Attention, and Frontoparietal Control Networks in Individuals with Chronic Traumatic Brain Injury
Source: J Int Neuropsychol Soc. 2016 Feb;22(2):263–79. doi: 10.1017/S1355617715001393 (PMC4763346; doi:10.1017/S1355617715001393)
Supplement: Supplementary file 1 [file S13556177150013935sup.zip › S1355617715001393sup006.pdf]

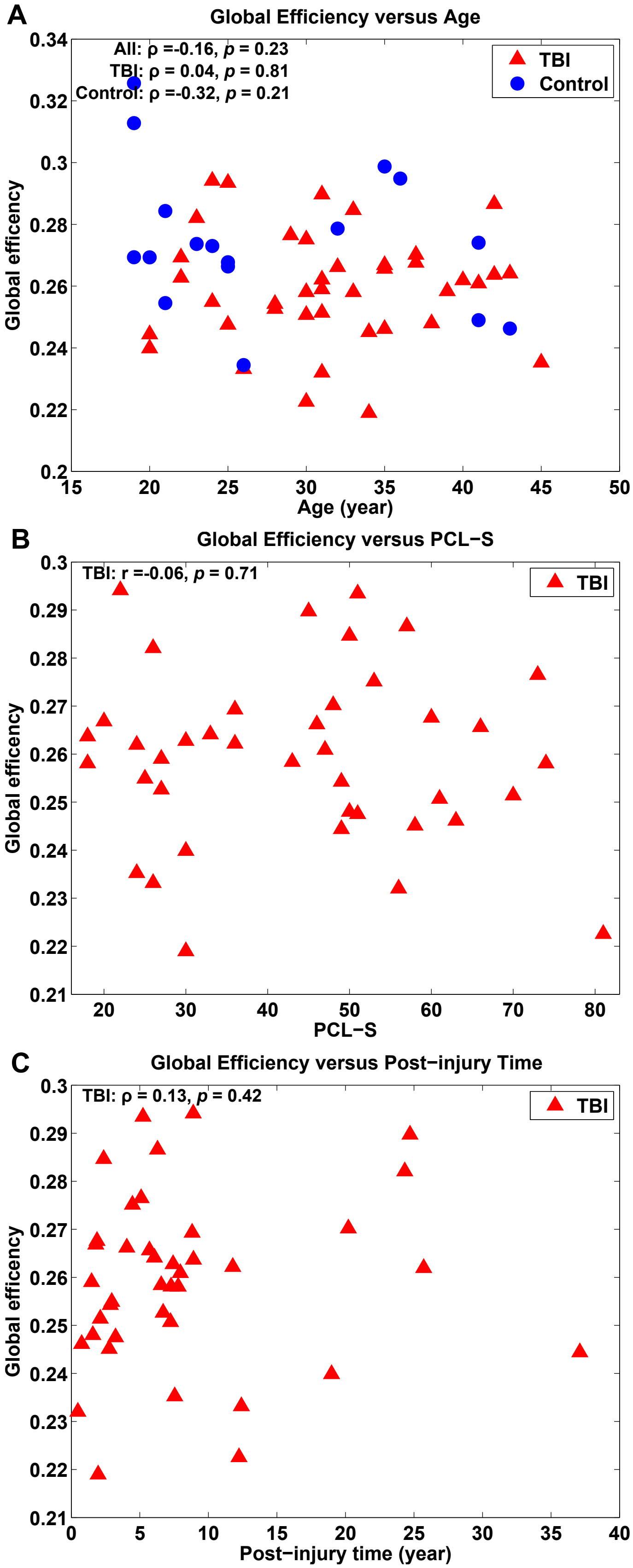

Fig. S2. Age (A), PCL-S (B) and post-injury time (C) of each participant versus the average global efficiency at network cost of 0.12, and age (D), PCL-S (E) and post-injury time (F) of each participant versus the average local efficiency at network cost of 0.15. The ρ-and r-values are the Spearman and Pearson correlation coefficients, respectively.
