# Supplementary material for: Disrupted Intrinsic Connectivity among Default, Dorsal Attention, and Frontoparietal Control Networks in Individuals with Chronic Traumatic Brain Injury
Source: J Int Neuropsychol Soc. 2016 Feb;22(2):263–79. doi: 10.1017/S1355617715001393 (PMC4763346; doi:10.1017/S1355617715001393)
Supplement: Supplementary file 1 [file S13556177150013935sup.zip › S1355617715001393sup007.pdf]

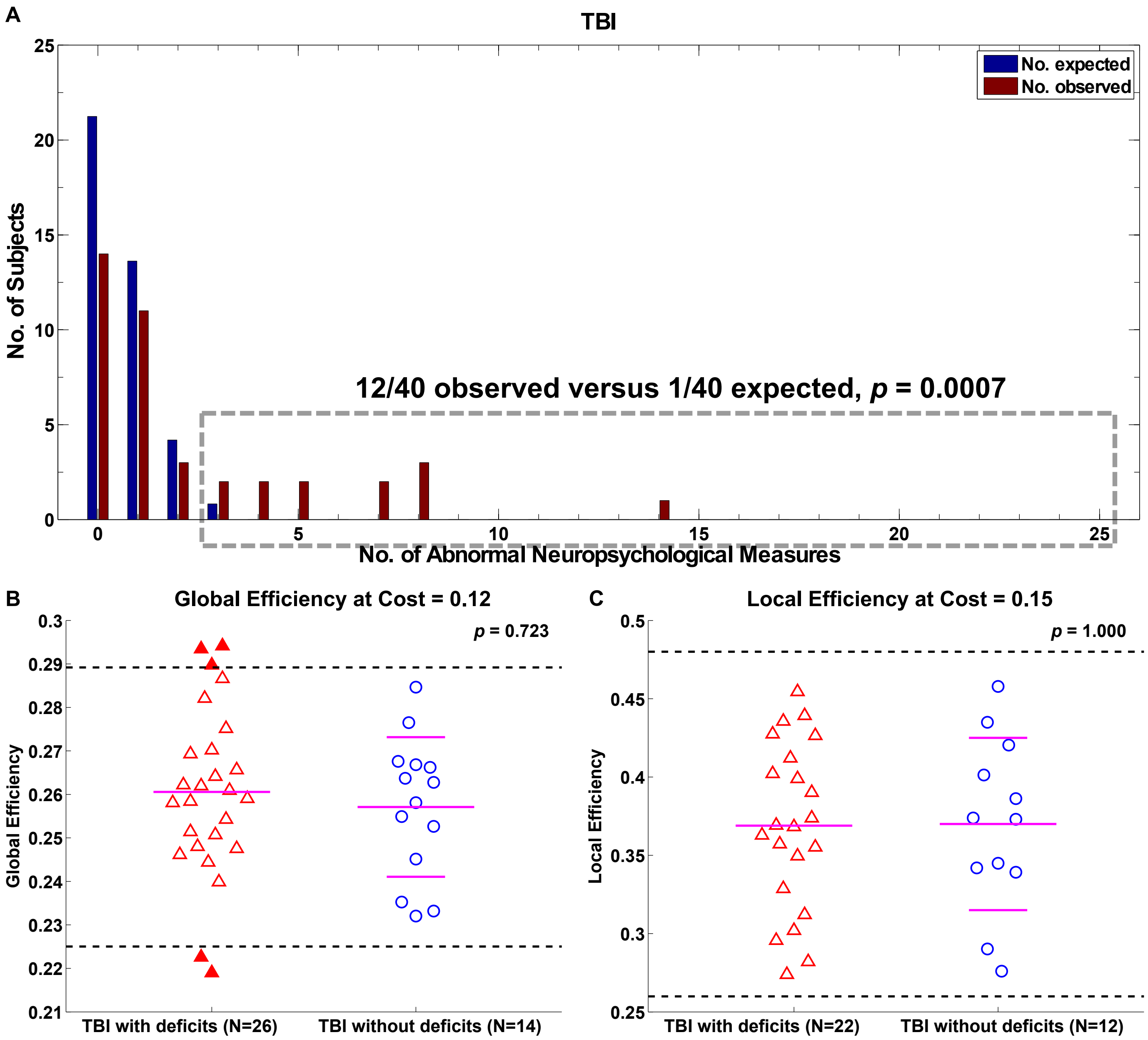

Fig. S3. Bar graph for observed and expected number of TBI individuals with ‘abnormally’ poor performance in neuropsychological tests relative to the controls (A), and scatter plots for global (B) and local efficiency (C) of TBI individuals with deficits in any of the 25 neuropsychological measures versus TBI individuals without any deficits in the 25 neuropsychological measures at network costs of 0.12 and 0.15, respectively. The 25 neuropsychological measures refer to all of the measures listed in Table 3 except current and premorbid IQs and the satisfaction with life scale. See Fig. 4 for the details of the scatter plots.
