# Supplementary material for: Disrupted Intrinsic Connectivity among Default, Dorsal Attention, and Frontoparietal Control Networks in Individuals with Chronic Traumatic Brain Injury
Source: J Int Neuropsychol Soc. 2016 Feb;22(2):263–79. doi: 10.1017/S1355617715001393 (PMC4763346; doi:10.1017/S1355617715001393)
Supplement: Supplementary file 1 [file S13556177150013935sup.zip › S1355617715001393sup008.pdf]

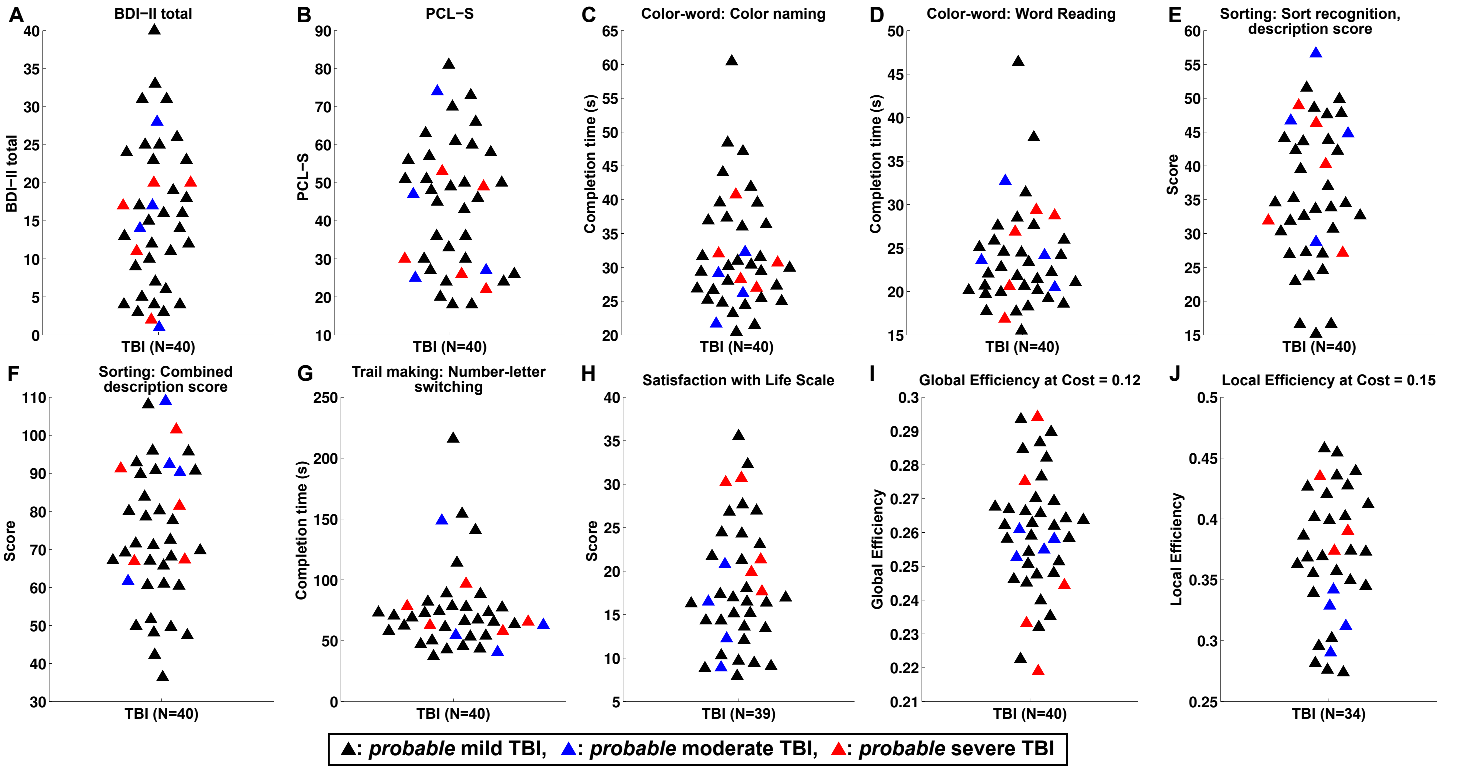

Fig. S4. Scatter plots for the BDI-II total (A), the PCL-S total (B), two sub-sets of the Color-Word Inference test (C-D), two sub-sets of the Card Sorting test (E-F), a sub-set of the Trail Making test (G), the Satisfaction with Life Scale (H), the global efficiency at the network cost of 0.12 (I) and the local efficiency at the network cost of 0.15 (J) of the TBI group according to estimated initial injury severity. The neuropsychological measures (C-H) were selected based on statistical significance of group comparions with the controls (p<0.1) in Table 3.
