# Supplementary material for: Disrupted Intrinsic Connectivity among Default, Dorsal Attention, and Frontoparietal Control Networks in Individuals with Chronic Traumatic Brain Injury
Source: J Int Neuropsychol Soc. 2016 Feb;22(2):263–79. doi: 10.1017/S1355617715001393 (PMC4763346; doi:10.1017/S1355617715001393)
Supplement: Supplementary file 1 [file S13556177150013935sup.zip › S1355617715001393sup009.pdf]

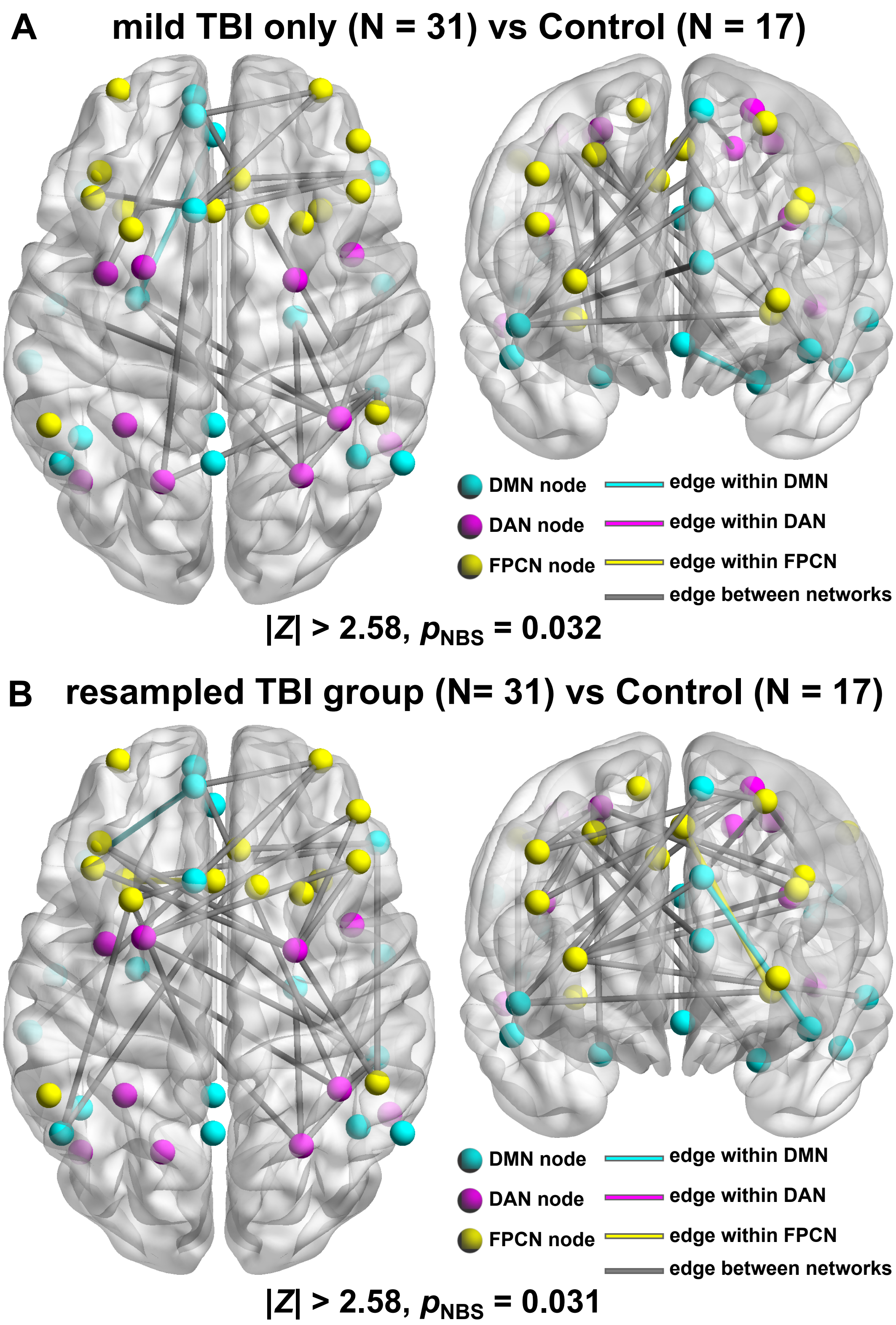

Fig. S5. An anatomical view of relatively reduced connectivity of the TBI subgroup comprising of individuals with *probable* mild TBI only (A) and one instance of resampled group by removing nine *probable* mild TBI participants from the original TBI group (B) at $|Z| > 2.58$, $p_{NBS}<0.05$. The average absolute value of Z-statistics for the group comparisons over the connections whose $|Z| >$ 2.58 of the selected, resampled group corresponds to the median among those of the entire 10,000 resampled pool.
