# Supplementary material for: Disrupted Intrinsic Connectivity among Default, Dorsal Attention, and Frontoparietal Control Networks in Individuals with Chronic Traumatic Brain Injury
Source: J Int Neuropsychol Soc. 2016 Feb;22(2):263–79. doi: 10.1017/S1355617715001393 (PMC4763346; doi:10.1017/S1355617715001393)
Supplement: Supplementary file 1 [file S13556177150013935sup.zip › S1355617715001393sup011.pdf]

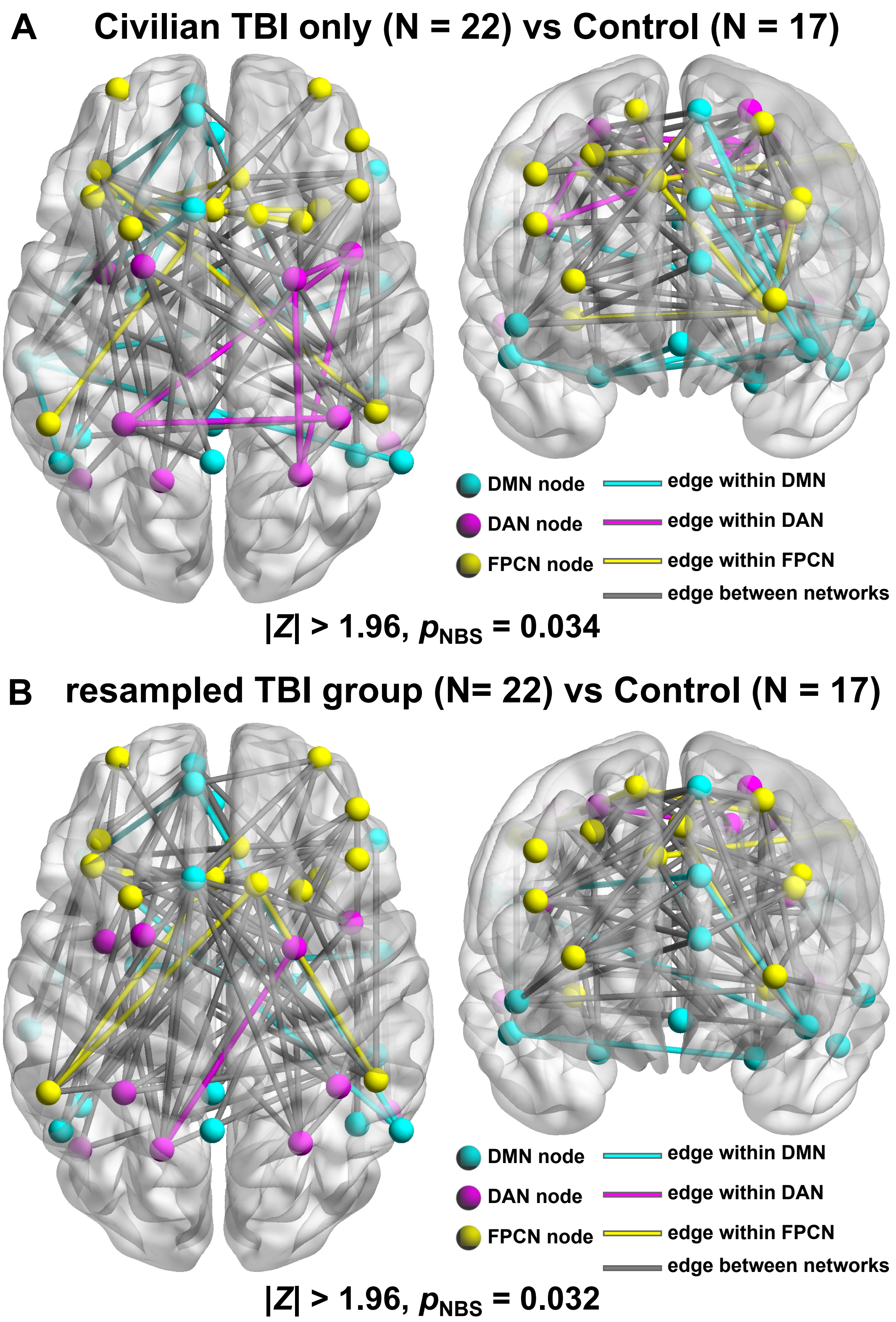

Fig. S7. An anatomical view of relatively reduced connectivity of the TBI subgroup comprising of individuals with civilian TBI only (A) and one instance of resampled group by removing the 18 civilians with TBI from the original TBI group (B) at $|Z| > 1.96$, $p_{NBS} < 0.05$. The average absolute value of Z-statistics for the group comparisons over the connections whose $|Z| > 1.96$ of the selected, resampled group corresponds to the median among those of the entire 10,000 resampled pool.
