# Supplementary material for: Disrupted Intrinsic Connectivity among Default, Dorsal Attention, and Frontoparietal Control Networks in Individuals with Chronic Traumatic Brain Injury
Source: J Int Neuropsychol Soc. 2016 Feb;22(2):263–79. doi: 10.1017/S1355617715001393 (PMC4763346; doi:10.1017/S1355617715001393)
Supplement: Supplementary file 1 [file S13556177150013935sup.zip › S1355617715001393sup012.pdf]

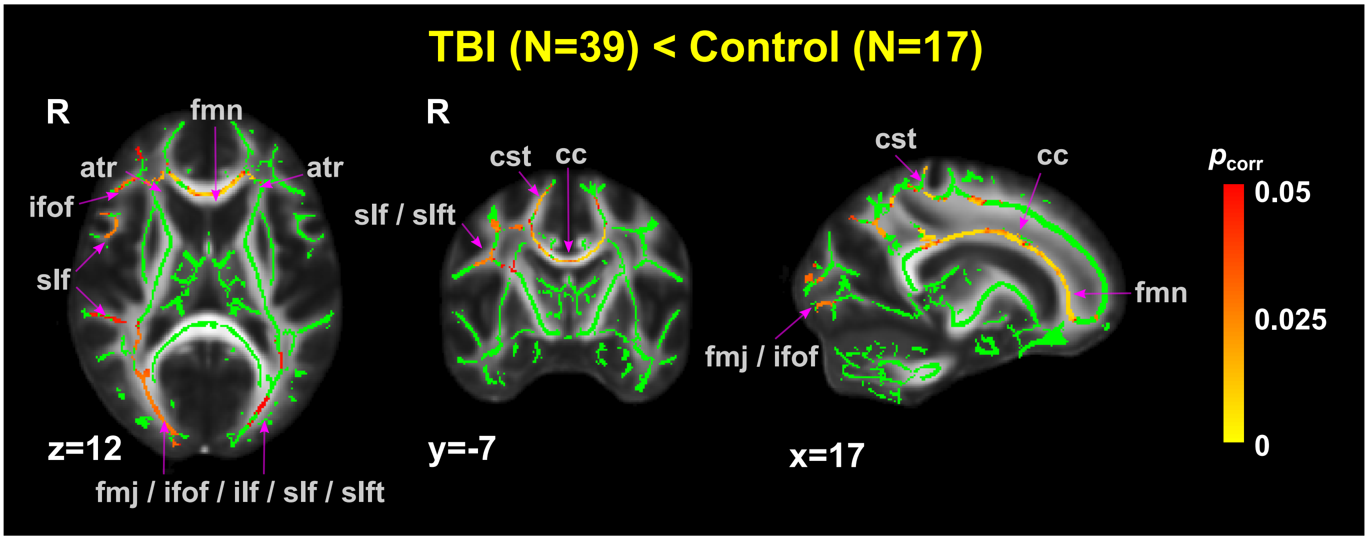

Fig. S8. Group comparisons of fractional anisotropy using tract-based spatial statistics (p_{corr} <0.05 with threshold-free cluster enhancement). The green lines are the skeletonized tracts drawn on the mean FA images obtained by averaging all FA maps registered to the FA template in the MNI 152 space. atr, anterior thalamic radiation; cc: corpus callosum (body); cst, corticospinal tract; fmj, forceps major; fmn, forceps minor; ifof, inferior fronto-occipital fasciculus; ilf, inferior longitudinal fasciculus; slf, superior longitudinal fasciculus; slft, superior longitudinal fasciculus (temporal).
