# Supplementary material for: Disrupted Intrinsic Connectivity among Default, Dorsal Attention, and Frontoparietal Control Networks in Individuals with Chronic Traumatic Brain Injury
Source: J Int Neuropsychol Soc. 2016 Feb;22(2):263–79. doi: 10.1017/S1355617715001393 (PMC4763346; doi:10.1017/S1355617715001393)
Supplement: Supplementary file 1 [file S13556177150013935sup.zip › S1355617715001393sup014.doc]

**Supplementary Material**

Title: Disrupted intrinsic connectivity among default, dorsal attention and frontoparietal control networks in individuals with chronic traumatic brain injury^†^

Authors: Kihwan Han^1,*^, Sandra B Chapman^1^, Daniel C Krawczyk^1,2^

Affiliations:

^1^Center for BrainHealth^®^ , School of Behavioral and Brain Sciences, The University of Texas at Dallas

^2^Department of Psychiatry, The University of Texas Southwestern Medical Center

Corresponding author: Kihwan Han, PhD

Center for BrainHealth^®^

School of Behavioral and Brain Sciences

2200 West Mockingbird Lane, Mail Stop: CBH

Dallas, TX 75235

USA

Email: kihwan.han@utdallas.edu

Tel: 1-972-883-3258

Fax: 1-214-905-3026

^†^The views and opinions expressed in this article are those of the authors and do not reflect the official policy or position of the Department of the Army, Department of the Air Force, Department of Defense or United States Government.

**SUPPLEMENTARY METHODS**

**Control analyses**

**Effects of demographics.** We assessed the effects of motion on our results by performing group comparisons of each subject's average FD after censoring and trimming and correlation analyses of the average FD after motion censoring and trimming versus the network measures for each of the groups. Since there were marginal groups differences in age, we assessed the effects of age on connectivity matrix by obtaining correlation coefficients between age and connectivity strengths across participants for each element of the connectivity matrix. Further, to assess potential effects of age on the efficiency measures (Achard & Bullmore, 2007), we performed correlation analyses of age versus the efficiency measures for each of the groups. We also assessed potential, residual effects of PTSD symptom severity on the efficiency measures by performing a correlation analysis of the PCL-S scores versus the efficiency measures for the TBI group. Due to a wide-range of post-injury times in the TBI participants, we also performed correlation analyses of post-injury time versus the efficiency measures for the TBI group.

**Neuropsychological deficits in the TBI group.** We assessed whether the TBI group had 'overall' deficits in the 25 neuropsychological measures (all the measures listed in Table 3 except current and premorbid IQ and the satisfaction with life scale) in the following manner. First, we defined that a TBI participant had a relative 'abnormality' in a neuropsychological measure if the given measure fell outside the two-standard-deviation-band compared to the mean of the controls (either above for the color-word and the trail making tests or below for the other measures). We then counted the number of relative 'abnormalities' in neuropsychological measures for each of the TBI participants. Lastly, we performed a one-sided Fisher's exact test to evaluate the number of TBI participants with relatively 'abnormal' neuropsychological performance in more than two measures versus the number expected by chance. The expected distribution of the number of TBI participants with relatively 'abnormal' neuropsychological performance is a binomial distribution with the probability that one neuropsychological measure shows 'abnormality' (either upper or lower tail of the normal distribution, i.e., *p≈*0.025). See Han et al. (2014) and Mac Donald et al. (2011) for details of this statistical procedure. To assess whether the presence of deficits in the neuropsychological measures in the TBI participants affected our findings, we compared the global and local efficiency of the TBI participants showing deficits in any of the 25 neuropsychological measures with those of TBI participants without any deficits.

**Effects of estimated initial injury severity.** Since *initial* injury severity of the TBI individuals ranged across *probable* mild, moderate and severe (see Discussion for the limitations regarding a mixture of TBI individuals with different estimated initial injury severity), we identified if there were any systematic effects of estimated initial injury severity on our findings as follows. First, we assessed the BDI-II and PCL-S total scores, six neuropsychological measures that showed noticeable group differences at *p*<0.1 (Table 3), and the global and local efficiency of the TBI individuals according to estimated initial injury severity. Next, we performed the NBS analysis with *probable* mild TBI individuals only (N=31). Further, we performed group connectivity matrix analyses by excluding nine (out of 31) *probable* mild TBI participants to match the sample size for the newly formed TBI group with that of the participants with *probable* mild TBI only. The goal of this analysis was to test whether the *probable* moderate and severe TBI participants contributed more to the connectivity differences than the *probable* mild TBI participants. To achieve this goal, we utilized a resampling method (10,000 instances of resampled groups) conceptually similar to the procedure described in Han & Talavage (2011). At each instance of the resampled groups, we performed group analyses of the connectivity matrices of a resampled TBI group and that of the healthy controls. Subsequently, we defined the magnitude of statistically significant group differences in connectivity by taking average absolute values of *Z*-statistics for the group comparison test over the connections with |*Z*|>2.58. We then assessed whether the magnitude of statistically significant group differences in connectivity for the *probable* mild TBI only group and the healthy control group fell outside the intervals of the 2.5th to the 97.5th percentiles (similar to 95% confidence intervals) for group differences, obtained from 10,000 resampled groups.

**Effect of depressive symptoms.** According to the BDI manual (Beck, Steer, & Brown, 1996), we first subdivided the TBI participants into two groups: a TBI with minimal depressive symptoms group (N=17; BDI<=13) and a TBI with mild to severe depressive symptom group (N=23; BDI>13). Next, we performed the NBS analysis with TBI individuals with minimal depressive symptoms only. Similar to the resampling analysis for the effects of estimated initial injury severity, we then performed group connectivity matrix analyses by selecting 17 (out of 23) TBI individuals with mild-to-severe depressive symptoms to match the sample size for the newly formed TBI group with that of the participants with TBI individuals with minimal depressive symptoms. Subsequently, we defined the magnitude of statistically significant group differences in connectivity by taking average absolute values of *Z*-statistics for the group comparison test over the connections with |*Z*|>1.96. We then assessed whether the magnitude of statistically significant group differences in connectivity for the TBI with minimal depressive symptoms group and the healthy control group fell outside the intervals of the 2.5th to the 97.5th percentiles (similar to 95% confidence intervals) for group differences, obtained from 10,000 resampled groups. Note that, in these NBS analyses, we did not incorporate the within-centered BDI-II scores covariates, which could retain potential effects of depressive symptoms on connectivity and increase group differences in connectivity. Lastly, we compared connectivity matrices, the global and local efficiency of the TBI participants with minimal depressive symptoms with those of TBI participants with mild to severe depressive symptoms.

**Effects of a mixture of civilians and veterans with TBI.** We first performed the NBS analysis with civilians with TBI only (N=22). Similar to the resampling analysis for the effects of estimated initial injury severity, we then performed group connectivity matrix analyses by excluding 18 (out of 22) civilians with TBI from the full TBI sample to match the sample size for the newly formed TBI group with that of the participants with TBI individuals with minimal depressive symptoms. Subsequently, we defined the magnitude of statistically significant group differences in connectivity by taking average absolute values of *Z*-statistics for the group comparison test over the connections with |*Z*|>1.96. We then assessed whether the magnitude of statistically significant group differences in connectivity for the civilian TBI only and the healthy control group fell outside the intervals of the 2.5th to the 97.5th percentiles (similar to 95% confidence intervals) for group differences, obtained from 10,000 resampled groups. Lastly, we compared connectivity matrices, the global and local efficiency of the veteran TBI group with those of the civilian TBI group.

**Whole-brain volumes.** To ascertain whether there were group differences in whole-brain volumes, whole-brain volumes were estimated using the SIENAX (Smith et al., 2002), within FSL software (Smith et al., 2004) then group comparisons were made controlling for age.

**Sustained DAIs.** To calculate DTI measures, we acquired diffusion-weighted volumes with gradients in 30 non-colinear directions with *b* value = 1000 s/mm^2^ and one *b*_0_ volume (TR/TE=4410/51ms, FA=90°, FOV=22.4x22.4cm; matrix=128x128; 50 slices, 2.0mm thick with 1.0 mm gap) from the 39 TBI individuals and 17 healthy controls in the same imaging session when these participants underwent resting-state fMRI and structural MRI scans. We then performed standard preprocessing procedures comprising of brain extraction and eddy-current correction and calculated fractional anisotropy (FA), using FSL software (Smith et al., 2004). The obtained FA values were analyzed using tract-based spatial statistic (TBSS; Smith et al., 2006) with the FMRIB58 FA template and a skeleton threshold of 0.2. For group comparisons, we conducted permutation test on skeletonized FA images using randomize with threshold-free cluster enhancement and 5000 permutations (Nichols & Holmes, 2001; Smith & Nichols, 2009). We then applied a thresholded of *p*<0.05 on the results to correct for multiple comparisons.

**Effects of an initial threshold level on NBS.** The effects of an initial threshold level for connectivity matrices on NBS results was assessed by applying additional threshold levels at *|Z|*>1.96 (*p*<0.05) and |*Z*|>2.81 (*p*<0.005), respectively.

**Partial correlation.** We further obtained a partial correlation for each pair of the regions, regressing out only positively correlated time-series from the other regions as in Spreng et al. (2013).

**Effects of global signal regression.** To identify the effects of global signal regression on our results, we additionally preprocessed our data without global signal regression and constructed connectivity matrices. Group analyses of the network measures were then performed.

**Effects of short distance nodes.** We performed the NBS analysis while retaining connectivity strengths between short distance nodes to identify whether our procedures introduced bias in our findings on disruptions in long-range connections.

**SUPPLEMENTARY RESULTS**

**Control analysis results**

**Effects of estimated initial injury severity.** There were no systematic effects of estimated *initial* injury severity on the TBI participants' BDI-II or PCL-S total scores, six selected neuropsychological measures, or global or local efficiency (Fig. S4). NBS analyses with mild TBI individuals only (Fig. S5A) essentially replicated the disruption patterns of the full TBI group that included all initial injury severity levels in Fig. 2. Again, NBS analyses with the resampled TBI sub-group, whose overall magnitude of statistically significant group differences in connectivity matrices corresponded to the median value among those of the entire resampled pool, essentially replicated the disruption patterns of the full TBI group (Fig S5B). The overall magnitude of statistically significant group differences in the connectivity matrices of the *probable* mild TBI sub-group versus the control group did not fall outside the 2.5th-to-97.5th percentile intervals of group differences obtained from 10,000 resampled groups. This suggests that there were no “significant” effects of estimated initial injury severity on the overall amount of statistically significant group differences in connectivity matrices.

**Effect of depressive symptoms.** NBS analyses with TBI individuals with minimal depressive symptoms (Fig. S6A) essentially replicated the disruption patterns of the full TBI group shown in Fig. 2. NBS analyses with the resampled TBI with depressive symptoms sub-group, essentially replicated the disruption patterns of the full TBI group (Fig S6B). The overall magnitude of statistically significant group differences in the connectivity matrices of the TBI with minimal depressive symptom group versus the control group fell within the 2.5th-to-97.5th percentile intervals of group differences obtained from 10,000 resampled groups. TBI with minimal depressive symptoms group and TBI with mild-to-severe depressive symptoms group did not show statistically significant group differences in connectivity matrices (*p*_NBS_>0.1) or efficiency measures (*p*>0.1) at network costs that were applied for the analyses with the full TBI sample.

**Effects of a mixture of civilians and veterans with TBI.** NBS analyses with 18 civilians with TBI (Fig. S7A) essentially replicated the disruption patterns of the full TBI group that included all spectrum depressive symptoms severity in Fig. 2. NBS analyses with the resampled TBI sub-group (18 veterans plus 4 civilians), essentially replicated the disruption patterns of the full TBI group (Fig S7B). The overall magnitude of statistically significant group differences in the connectivity matrices of the civilian TBI group versus the control group fell within the 2.5th-to-97.5th percentile intervals of group differences obtained from 10,000 resampled groups. The civilian TBI and resampled TBI groups did not show statistically significant group differences in connectivity matrices (*p*_NBS_>0.1) or efficiency measures (*p*>0.1) at network costs that were applied for the analyses with the full TBI sample.

**REFERENCES**

Achard, S., & Bullmore, E. (2007). Efficiency and cost of economical brain functional networks. *PLoS Computational Biology*, *3*, e17. doi:10.1371/journal.pcbi.0030017

Han, K., Mac Donald, C.L., Johnson, A.M., Barnes, Y., Wierzechowski, L., Zonies, D., … Brody, D.L. (2014). Disrupted modular organization of resting-state cortical functional connectivity in U.S. military personnel following concussive “mild” blast-related traumatic brain injury. *Neuroimage*, *84*, 76–96.

Han, K., & Talavage, T.M. (2011). Effects of combining field strengths on auditory functional MRI group analysis: 1.5T and 3T. *Journal of Magnetic Resonance Imaging*, *34*, 1480–1488.

Mac Donald, C.L., Johnson, A.M., Cooper, D., Nelson, E.C., Werner, N.J., Shimony, J.S., … Brody, D.L. (2011). Detection of blast-related traumatic brain injury in U.S. military personnel. *New England Journal of Medicine*, *364*, 2091–2100.

Nichols, T.E., & Holmes, A.P. (2001). Nonparametric permutation tests for functional neuroimaging: A primer with examples. *Hum Brain Mapp*, *15*, 1–25.

Smith, S.M., Jenkinson, M., Johansen-Berg, H., Rueckert, D., Nichols, T.E., Mackay, C.E., … Behrens, T.E.J. (2006). Tract-based spatial statistics: Voxelwise analysis of multi-subject diffusion data. *NeuroImage*, *31*, 1487–1505.

Smith, S.M., Jenkinson, M., Woolrich, M.W., Beckmann, C.F., Behrens, T.E.J., Johansen-Berg, H., … Matthews, P.M. (2004). Advances in functional and structural MR image analysis and implementation as FSL. *Neuroimage*, *23*, S208–S219.

Smith, S.M., & Nichols, T.E. (2009). Threshold-free cluster enhancement: Addressing problems of smoothing, threshold dependence and localisation in cluster inference. *NeuroImage*, *44*, 83–98.

Smith, S.M., Zhang, Y., Jenkinson, M., Chen, J., Matthews, P.M., Federico, A., & De Stefano, N. (2002). Accurate, robust, and automated longitudinal and cross-sectional brain change analysis. *Neuroimage*, *17*, 479–489.

Spreng, R.N., Sepulcre, J., Turner, G.R., Stevens, W.D., & Schacter, D.L. (2013). Intrinsic architecture underlying the relations among the default, dorsal Attention, and frontoparietal control networks of the human brain. *Journal of Cognitive Neuroscience*, *25*, 74–86.

**SUPPLEMENTARY FIGURE LEGENDS**

**Fig. S1.** Average framewise displacement (FD) of each participant after motion censoring and trimming versus the average global (A) and local (B) efficiency at network costs of 0.12 and 0.15, respectively. The r-values are the Pearson correlation coefficients.

**Fig. S2.** Age (A), PCL-S (B) and post-injury time (C) of each participant versus the average global efficiency at network cost of 0.12, and age (D), PCL-S (E) and post-injury time (F) of each participant versus the average local efficiency at network cost of 0.15. The ρ-and r-values are the Spearman and Pearson correlation coefficients, respectively.

**Fig. S3.** Bar graph for observed and expected number of TBI individuals with ‘abnormally’ poor performance in neuropsychological tests relative to the controls (A), and scatter plots for global (B) and local efficiency (C) of TBI individuals with deficits in any of the 25 neuropsychological measures versus TBI individuals without any deficits in the 25 neuropsychological measures at network costs of 0.12 and 0.15, respectively. The 25 neuropsychological measures refer to all of the measures listed in Table 3 except current and premorbid IQs and the satisfaction with life scale. See Fig. 4 for the details of the scatter plots.

**Fig. S4.** Scatter plots for the BDI-II total (A), the PCL-S total (B), two sub-sets of the Color-Word Inference test (C-D), two sub-sets of the Card Sorting test (E-F), a sub-set of the Trail Making test (G), the Satisfaction with Life Scale (H), the global efficiency at the network cost of 0.12 (I) and the local efficiency at the network cost of 0.15 (J) of the TBI group according to estimated initial injury severity. The neuropsychological measures (C-H) were selected based on statistical significance of group comparisons with the controls (p<0.1) in Table 3.

**Fig. S5.** An anatomical view of relatively reduced connectivity of the TBI subgroup comprising of individuals with probable mild TBI only (A) and one instance of resampled group by removing the nine probable mild TBI participants from the original TBI group (B) at |*Z*| > 2.58, *p*_NBS_<0.05. The average absolute value of *Z*-statistics for the group comparisons over the connections whose |*Z*| > 2.58 of the selected, resampled group corresponds to the median among those of the entire 10,000 resampled pool.

**Fig. S6.** An anatomical view of relatively reduced connectivity of the TBI subgroup comprising of TBI individuals with minimal depressive symptoms only (A) and one instance of resampled group by selecting the same number of TBI individuals with mild-to-severe depressive symptoms from the original TBI group (B) at |*Z*| > 1.96, *p*_NBS_<0.1. The average absolute value of *Z*-statistics for the group comparisons over the connections whose |*Z*| > 1.96 of the selected, resampled group corresponds to the median among those of the entire 10,000 resampled pool.

**Fig. S7.** An anatomical view of relatively reduced connectivity of the TBI subgroup comprising of individuals with civilian TBI only (A) and one instance of resampled group by removing the 18 civilians with TBI from the original TBI group (B) at |*Z*| > 1.96, *p*_NBS_<0.05. The average absolute value of Z-statistics for the group comparisons over the connections whose |*Z*| > 1.96 of the selected, resampled group corresponds to the median among those of the entire 10,000 resampled pool.

**Fig. S8.** Group comparisons of fractional anisotropy using tract-based spatial statistics (*p*_corr_ <0.05 with threshold-free cluster enhancement). The green lines are the skeletonized tracts drawn on the mean FA images obtained by averaging all FA maps registered to the FA template in the MNI 152 space. atr, anterior thalamic radiation; cc: corpus callosum (body); cst, corticospinal tract; fmj, forceps major; fmn, forceps minor; ifof, inferior fronto-occipital fasciculus; ilf, inferior longitudinal fasciculus; slf, superior longitudinal fasciculus; slft, superior longitudinal fasciculus (temporal).

**Fig. S9.** An anatomical view of reduced connectivity in TBI relative to the controls at |*Z*|>1.96 (A) and |*Z*|>2.81 (B). The left side is the left hemisphere.

**Fig. S10.** Group comparisons of average connectivity matrices based on partial correlation coefficients. (A): Average connectivity of the TBI group. (B): Average connectivity of the control group. (C): Histogram for *Z*-statistics of group comparisons on average connectivity. (D) Thresholded *Z*-statistic map for group comparisons (*p*_NBS_<0.05 at |*Z*|>1.96). Colorbars in (A) and (B) represent Fisher's *Z*-transformed correlation coefficients.

**Fig. S11.** An anatomical view of reduced (A) and elevated (B) connectivity in TBI relative to the controls (*p*_NBS_<0.05 at |*Z*|>1.96) based on partial correlation coefficients. The left side is the left hemisphere.

**Fig. S12.** Global, local and cost efficiency of the TBI group and the controls without global signal regression. (A)-(C): Average global, local and cost efficiency as a function of network cost, respectively. Note that, to reliably perform group analyses, we limited ranges of network cost (from 0.01 to 0.44 in step size of 0.01) to include N≥5 per group. (D), (E): Scatter plots for global and local efficiency at network costs of 0.12 and 0.16, respectively. See Fig. 4 for the details of the scatter plots.

**Fig. S13.** The number of reduced connections in TBI relative to the controls (thresholded at |*Z*|

>1.96, *p*_NBS_<0.05) by distance between nodes while retaining connectivity strengths between short distance nodes (< 20 mm).
